# Network topology of symbolic and nonsymbolic number comparison

**DOI:** 10.1162/netn_a_00144

**Published:** 2020-08-01

**Authors:** Benjamin N. Conrad, Eric D. Wilkey, Darren J. Yeo, Gavin R. Price

**Affiliations:** Psychology and Human Development, Vanderbilt University, Nashville, TN, USA; Vanderbilt Brain Institute, Vanderbilt University, Nashville, TN, USA; Psychology and Human Development, Vanderbilt University, Nashville, TN, USA; Vanderbilt Brain Institute, Vanderbilt University, Nashville, TN, USA; Brain & Mind Institute, Western University, London, ON, Canada; Psychology and Human Development, Vanderbilt University, Nashville, TN, USA; Vanderbilt Brain Institute, Vanderbilt University, Nashville, TN, USA; Division of Psychology, School of Social Sciences, Nanyang Technological University, Singapore; Psychology and Human Development, Vanderbilt University, Nashville, TN, USA; Vanderbilt Brain Institute, Vanderbilt University, Nashville, TN, USA

**Keywords:** Numerical cognition, Functional connectivity, Modularity, Community allegiance, Beta-series correlation

## Abstract

Studies of brain activity during number processing suggest symbolic and nonsymbolic numerical stimuli (e.g., Arabic digits and dot arrays) engage both shared and distinct neural mechanisms. However, the extent to which number format influences large-scale functional network organization is unknown. In this study, using 7 Tesla MRI, we adopted a network neuroscience approach to characterize the whole-brain functional architecture supporting symbolic and nonsymbolic number comparison in 33 adults. Results showed the degree of global modularity was similar for both formats. The symbolic format, however, elicited stronger community membership among auditory regions, whereas for nonsymbolic, stronger membership was observed within and between cingulo-opercular/salience network and basal ganglia communities. The right posterior inferior temporal gyrus, left intraparietal sulcus, and two regions in the right ventromedial occipital cortex demonstrated robust differences between formats in terms of their community membership, supporting prior findings that these areas are differentially engaged based on number format. Furthermore, a unified fronto-parietal/dorsal attention community in the nonsymbolic condition was fractionated into two components in the symbolic condition. Taken together, these results reveal a pattern of overlapping and distinct network architectures for symbolic and nonsymbolic number processing.

## Author Summary

Previous studies of local activity levels suggest that both shared and distinct neural mechanisms support the processing of symbolic (Arabic digits) and nonsymbolic (dot sets) number stimuli, involving regions distributed across frontal, temporal, and parietal cortices. Network-level characterizations of functional connectivity patterns underlying number processing have gone unexplored, however. In this study we examined the whole-brain functional architecture of symbolic and nonsymbolic number comparison. Stronger community membership was observed among auditory regions during symbolic processing, and among cingulo-opercular/salience and basal ganglia networks for nonsymbolic. A dual versus unified fronto-parietal/dorsal attention community organization was observed for symbolic and nonsymbolic formats, respectively. Finally, the inferior temporal gyrus and left intraparietal sulcus, both thought to be preferentially involved in processing number symbols, demonstrated robust differences in community membership between formats.

## INTRODUCTION

The representation and manipulation of numerical information in the brain has received considerable attention over the last 20 years. A longstanding debate in the field regards the extent to which symbolic (e.g., Arabic digits) and nonsymbolic (e.g., dot arrays) number formats engage shared versus distinct neural mechanisms (Cohen Kadosh & Walsh, 2009; Knops, 2017; Leibovich & Ansari, 2016; Reynvoet & Sasanguie, 2016). Regions of the bilateral intraparietal sulcus (IPS) have been found to be involved in symbolic (Ansari et al., 2005; Eger, Sterzer, Russ, Giraud, & Kleinschmidt, 2003; Holloway, Battista, Vogel, & Ansari, 2013; Kaufmann et al., 2005; Pinel, Dehaene, Riviére, & Le Bihan, 2001) and nonsymbolic (Ansari & Dhital, 2006; Cantlon, Brannon, Carter, & Pelphrey, 2006; Piazza, Izard, Pinel, Le Bihan, & Dehaene, 2004) numerical processing during both adaptation and number comparison paradigms, supporting proposals that the IPS provides a substrate for quantity encoding shared between formats (Dehaene & Cohen, 1995; Dehaene, Piazza, Pinel, & Cohen, 2003). A recent meta-analysis of the extant functional magnetic resonance imaging (fMRI) literature supports the notion that the parietal lobe subserves a shared representation of numerical magnitude across nonsymbolic and symbolic formats, detecting convergence of activation foci between symbolic and nonsymbolic number processing in several bilateral parietal areas, as well as medial superior frontal gyrus (Sokolowski, Fias, Mousa, & Ansari, 2016). Such findings suggest shared neural resources for numerical processing across formats.

In addition to evidence for shared neural substrates across numerical formats, there is also considerable evidence for distinct mechanisms between formats. Symbolic number comparison has been shown, for instance, to engage left superior temporal gyrus (STG) (Holloway, Price, & Ansari, 2010) as well as bilateral angular gyri, to a greater extent than nonsymbolic number comparison, potentially reflecting increased reliance on verbal mechanisms during number symbol processing (Ansari, Lyons, van Eimeren, & Xu, 2007; Dehaene et al., 2003; He, Zuo, Chen, & Humphreys, 2014; Holloway et al., 2010). A meta-analytic contrast between formats suggests a general asymmetry in parietal activations, such that Arabic digits preferentially engage left inferior parietal cortex, whereas nonsymbolic stimuli more strongly engage distributed areas of right superior parietal, right frontal, and insular cortices (Sokolowski et al., 2016). Symbolic number processing has also been shown to engage ventral occipitotemporal (vOT) areas involved in the decoding of visual symbols (Dehaene & Cohen, 1995), such as during active processing of Arabic digits (Grotheer, Herrmann, et al., 2016; Pollack & Price, 2019; Shum et al., 2013; Yeo, Wilkey, & Price, 2017). However, it is unclear the extent to which vOT areas are differentially engaged between symbolic and nonsymbolic number formats. For example, the meta-analytic contrast performed by Sokolowski et al. (2016) found no clusters in the vOT. In summary, the current state of evidence suggests that symbolic and nonsymbolic number processing share neural resources in frontal and parietal regions, but also engage distinct mechanisms distributed across the frontal, parietal, and temporal cortices.

While prior work has focused on the extent to which brain regions are similarly or differentially activated across number formats, complementary questions remain in regards to how these regions interact and participate in functional networks during number processing. Since the earliest theories of numerical cognition, there has been an implicit understanding that multiple brain systems and subnetworks are involved in extracting and operating on numerical information (Campbell, 1994; Dehaene, 1992; McCloskey, 1992; McCloskey, Caramazza, & Basili, 1985), yet to date, whole-brain network-level descriptions have gone unexplored. We propose that description of symbolic and nonsymbolic number processing mechanisms in terms of their widespread functional interactions among multiple brain areas and systems, such as those implicated in visual perception, attention, and cognitive control, will shed new light on the format-dependent versus format-independent processing debate, and provide complementary insights into how numerical cognition is accomplished in the human brain.

Graph theory provides a methodological framework for characterizing complex networks, such as those derived from whole-brain functional connectivity data (Bassett & Gazzaniga, 2011; Bassett & Sporns, 2017). One application that has been particularly fruitful in cognitive neuroscience has been the use of graph topology–based clustering techniques to group brain regions into functional subnetworks, which can then be associated with various cognitive and behavioral functions (Garcia, Ashourvan, Muldoon, Vettel, & Bassett, 2018; Power et al., 2011; Sporns & Betzel, 2016). In the present study, we collected 7 Tesla fMRI data in adults who performed a numerical magnitude comparison task involving symbolic and nonsymbolic stimuli. We employed task-evoked functional connectivity (i.e., beta-series correlation; Rissman, Gazzaley, & DŠEsposito, 2004) and modularity maximization–based clustering (Lancichinetti & Fortunato, 2012) to assess the network topology, or organization, of symbolic and nonsymbolic number comparison. We contrasted topologies between formats first at the macroscale (i.e., global level involving all regions), then the community level (i.e., involving groups of regions), and finally the individual region level. Specifically, we asked the following questions: (1) Is there a difference in the whole-brain functional connectivity structure (i.e., global modularity) between the symbolic and nonsymbolic formats? (2) What is the topographic layout of functional communities in response to each format? (3) To what extent is the connectivity within and between each community significantly different between formats? (4) Do any regions demonstrate a significant change in their whole-brain connectivity profile between formats?

Given that the same task was performed across all trials, we expected that multiple task-level processes would be shared between formats, for example, involving cognitive control and response selection mechanisms. We thus predicted a similar macroscale network architecture would be observed for each format (Krienen, Yeo, & Buckner, 2014), indicated by an equivalent degree of global modularity, and the inclusion of a fronto-parietal network involving IPS and prefrontal regions. We hypothesized, however, that differences would arise among regional communities, with the symbolic format differentially engaging ventral visual and temporoparietal pathways involved in object recognition and orthographic decoding, and the nonsymbolic format engaging an occipitoparietal pathway involved in object location, individuation, and summation coding. Furthermore, if the posterior inferior temporal gyrus (pITG) is preferentially involved in the recognition of Arabic numerals, we expected this region to show increased interaction with parietal regions. However, if the pITG plays a nonspecific role in number processing more generally, we expected a similar pattern between formats in the functional interactions of this region.

## RESULTS

### Behavioral Results

Descriptive statistics of behavioral performance are provided in Table 1. Mean reaction times were computed from correctly answered trials only. Symbolic comparison trials elicited a significantly lower error rate than nonsymbolic trials using paired tests (*t*(32) = 2.20, *p* = 0.035). A significant difference in mean reaction time was also observed, whereby nonsymbolic trials required approximately 45 ms more time to complete than symbolic trials (*t*(32) = −6.27, *p* = 5.02 ×10^−7^). In sum, participants were both faster and more accurate when solving symbolic versus nonsymbolic comparisons.

**Table 1.** Scanner task performance.

	Mean	Standard deviation	Minimum	Maximum
Symbolic condition accuracy (%)	97.1	3.7	85.0	100.0
Nonsymbolic condition accuracy (%)	94.9	7.0	71.3	100.0
Symbolic condition reaction time (ms)	774.2	142.6	550.9	1,133.6
Nonsymbolic condition reaction time (ms)	818.8	129.0	627.1	1,130.2

### Global Modularity Across Topological Scales

**Figure 1.**
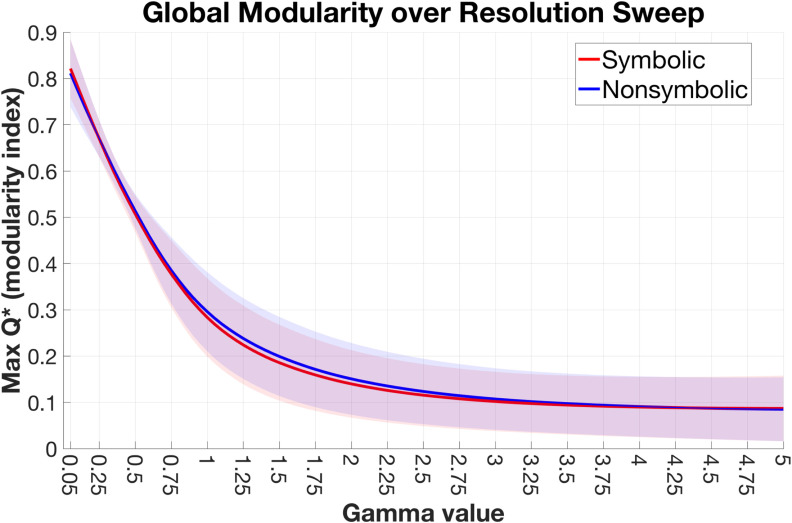
No format differences in global modularity. The maximum global modularity (Q*) value for each participant/format was extracted at every step along the resolution sweep (*γ* = 0.05–5.0, with steps of 0.05). The mean lines are plotted along with shaded bars representing the standard deviation across participants.

Our first question was, is there a difference in overall modular organization between formats? The modularity of a network is indexed by the statistic, Q*, and provides a summary measure of the extent to which a network can be decomposed into nonoverlapping communities when eachnode(i.e., region) is assigned to only one community (see Methods for algorithmic definition). A positive Q* value indicates that intracommunity connections are stronger than chance and thus, Q* summarizes the modular topology of a network in regards to the relative balance of segregated versus integrated connectivity structure (high versus low Q*, respectively) (Newman, 2006). For each subject, we extracted the max Q* observed over 100 iterations for the symbolic and nonsymbolic networks separately, and performed this procedure at each step of the structural resolution parameter (*γ*) over the resolution range of interest (Figure 1; see Methods for more details on this range). A paired *t* test was performed at each step and revealed no significant difference between the max Q* in the symbolic versus nonsymbolic conditions (*p* > 0.05 uncorrected). This result indicates that there was no consistent difference between formats at any topological scale in the degree of global segregation/integration across the brain.

### Community Topology

While our first analysis indicated a similar modular structure between formats at the global level, similar macroscale characteristics can arise from differing underlying topologies, making it important to supplement global metrics with more fine-grained assessment of specific network elements (Sporns, 2014). Therefore, we investigated the subnetwork organization of functional communities, looking at community topography for each format separately and differences between formats in the degree of community allegiance.

#### Functional communities within format

The graph-theoretical concept of allegiance refers to the probability of two regions being assigned to the same community across multiple partitions of a network, and has been used extensively to study community organization during task performance (Bassett, Yang, Wymbs, & Grafton, 2015; Bertolero, Thomas Yeo, & D’Esposito, 2015; Braun et al., 2015; Chai, Mattar, Blank, Fedorenko, & Bassett, 2016; Mattar, Cole, Thompson-Schill, & Bassett, 2015; Rizkallah et al., 2018; Telesford et al., 2016). The allegiance matrix contains the probability for every region pair, that is, the tendency for two brain regions to be part of the same functional community, and thus describes region-to-region associations with respect to shared community membership. Where functional connectivity asks of two regions, “How much do they communicate?,” allegiance asks, “Do they belong to the same group?” While the two metrics are related (and especially so in functional connectivity matrices due to the transitive nature of time series correlations), they can in principle diverge in a number of scenarios. For instance, two regions may exhibit relatively low connectivity to each other but, due to stronger association with other members of the same community, demonstrate high allegiance. In another case, a hub region with strong connectivity to two communities may demonstrate relatively low allegiance to any one region, due to inconsistency in its community assignment (i.e., it belongs equally to two communities). From these examples, one can appreciate that allegiance and connectivity provide complementary information about relationships among nodes of a network. Importantly, however, an allegiance-based approach has some advantages. As an allegiance matrix is derived from network partitions (which reduce connectivity information down to singular community assignments), allegiance values are robust to (1) noise inherent in connectivity measurements, as well as (2) differences in raw connectivity strengths and/or distributions across conditions or subjects, making them appealing for studying functional reconfiguration across task states (Bassett et al., 2015). In other words, the allegiance matrix serves as a pure representation of a network’s modular topology. Indeed, an allegiance-based approach was shown to be more sensitive to differences in functional network architecture compared with connectivity information alone (Bassett et al., 2015). In this study we used group-level allegiance measures to assess community and region-level topology during symbolic and nonsymbolic number comparison (see Methods).

**Figure 2.**
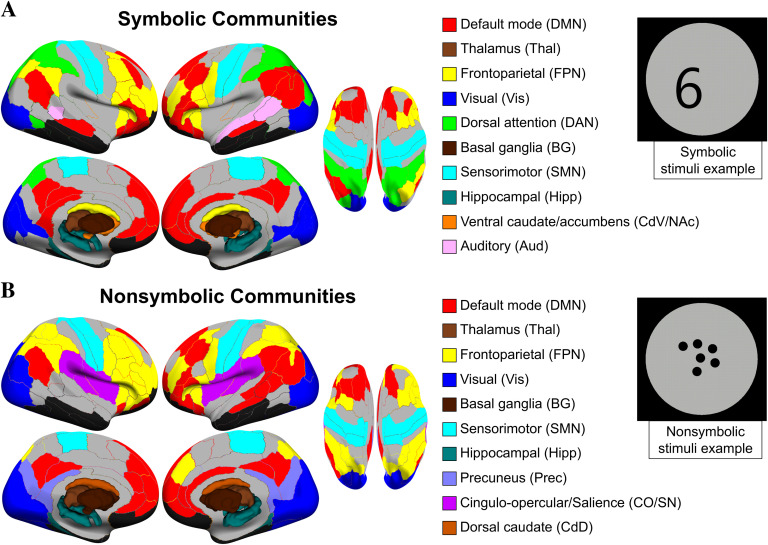
Functional communities by format. Group-level communities were determined for symbolic and nonsymbolic formats separately. The depicted communities represent the reduced set that passed our selection criterion of showing strong modularity in subject-level connectivity matrices (see Supporting Information Methods and Supporting Information Figure S1 for details on the selection procedure). Cortical regions are plotted to a surface representation and subcortical regions are presented as 3D volumes. Community labels were determined based on voxel-wise overlap with the networks delineated in Power et al. (2011), or, when an analogous network did not exist in Power et al., based on the anatomical region(s). Colors were chosen to match those networks where possible. Gray indicates regions that did not belong to a selected community; black indicates regions excluded based on signal dropout (see Supporting Information Methods).

To evaluate community-level organization, one must first define the topological “resolution” of interest, which relates to the size of communities detected via modularity maximization and is modulated by the resolution parameter *γ* (Reichardt & Bornholdt, 2006). Since our goal was to compare topologies between the symbolic and nonsymbolic networks, we sought a resolution at which there was a maximal balance between similarities and differences in group-level community structure between formats (Mattar et al., 2015). Based on this heuristic, we found an optimal resolution at *γ* = 2.45 and used this value for subsequent community-level analyses (see Methods for details on this procedure). Our group-level consensus clustering procedure delineated 44 communities for symbolic (25 nonsingleton and 19 singleton, where singleton refers to a community of just one region) and 36 communities for nonsymbolic (23 nonsingleton, 13 singleton). We reduced the set of communities to only those that demonstrated a strong modularity contribution (*Q*_c_*) at the subject level, ensuring that the communities of interest were (1) not driven by noise, and (2) not solely a feature of the group-level networks, but biologically meaningful in individuals (Bassett, Owens, Daniels, & Porter, 2012; Betzel et al., 2017; Guimerà, Sales-Pardo, & Amaral, 2004). Briefly, we computed the *Q*_c_* for each community in the subject-level connectivity matrices and compared against null models, selecting for further analyses only those that exceeded the 99th percentile of the null distribution (see Supporting Information Methods and Supporting Information Results Figure S1), in an approach adapted from Betzel et al. (2017). This procedure resulted in 10 of 25 communities selected for symbolic and 10 of 23 communities selected for nonsymbolic (Figure 2).

One challenge in interpreting functional brain network results based on data-driven clustering solutions is the assignment of labels to the resulting communities (Yeo et al., 2011). Using the widely adopted Power et al. (2011) network definitions, we took a quantitative approach in which we calculated the voxel-wise overlap of our final community assignments with the consensus assignments from Power et al. (acquired from https://www.jonathanpower.net/2011-neuron-bigbrain.html). In almost all cases where an analogous community existed in the Power et al. partition, a clear “winner” was determined based on the relative percentage overlap versus other communities, including the default mode network (DMN), fronto-parietal task control network (FPN), dorsal attention network (DAN), sensorimotor network (SMN), visual (Vis), auditory (Aud), hippocampus (Hipp), thalamus (Thal), and basal ganglia (BG) communities. In the case of our smaller communities, involving the dorsal caudate (CdD), ventral caudate and nucleus accumbens (CdV/NAc), and precuneus (Prec), there was not a corresponding Power et al. assignment, so we labeled these according to their anatomical description in the Brainnetome atlas (see Methods and Supporting Information Table S1) (Fan et al., 2016). One nonsymbolic community, involving primarily bilateral insular regions, was composed equally of voxels within the cingulo-opercular task control (CO, 40.1%) and salience network (SN, 39.9%) communities from Power et al. Given that a clear distinction could not be made in this case, we refer to this community by using the combined label CO/SN.

Qualitative differences were observed between formats in the existence and topography of individual communities (Figure 2). The FPN community, for instance, was more distributed in the nonsymbolic condition, including bilateral superior and inferior parietal regions as well as a majority of prefrontal regions in the right hemisphere. For symbolic, the regions making up the FPN in nonsymbolic were largely divided into a more ventral FPN and a DAN community, with the DAN including superior parietal lobule (SPL) regions and a region of right pITG (see next section and Supporting Information Figure S2 for further investigation of the dual versus unified FPN/DAN network architecture between formats). Furthermore, a left-lateralized auditory (Aud) community, including left STG and bilateral superior temporal sulcus regions, was only detected in the symbolic condition, and a CO/SN community, involving primarily bilateral insular regions, was only detected in the nonsymbolic condition. On the other hand, the visual, sensorimotor, default mode, and subcortical communities were topographically comparable between formats. Supporting Information Table S1 includes labels and community assignments for all regions of the Brainnetome Atlas.

As a final analysis of community topography, we sought to determine whether the observed dual versus unified FPN/DAN network architecture in the symbolic and nonsymbolic conditions, respectively, was specific to the particular *γ* value employed in these analyses (i.e., *γ* = 2.45), or was robust across *γ* values. The dual FPN/DAN network architecture in the symbolic condition persisted across a relatively large range of *γ* (Supporting Information Figure S2), whereas these communities were not consistently distinguished in the nonsymbolic condition, suggesting that the observed difference in FPN/DAN integration between formats is robust across *γ* values.

#### Differences in the degree of within- and between-community allegiance between formats

**Figure 3.**
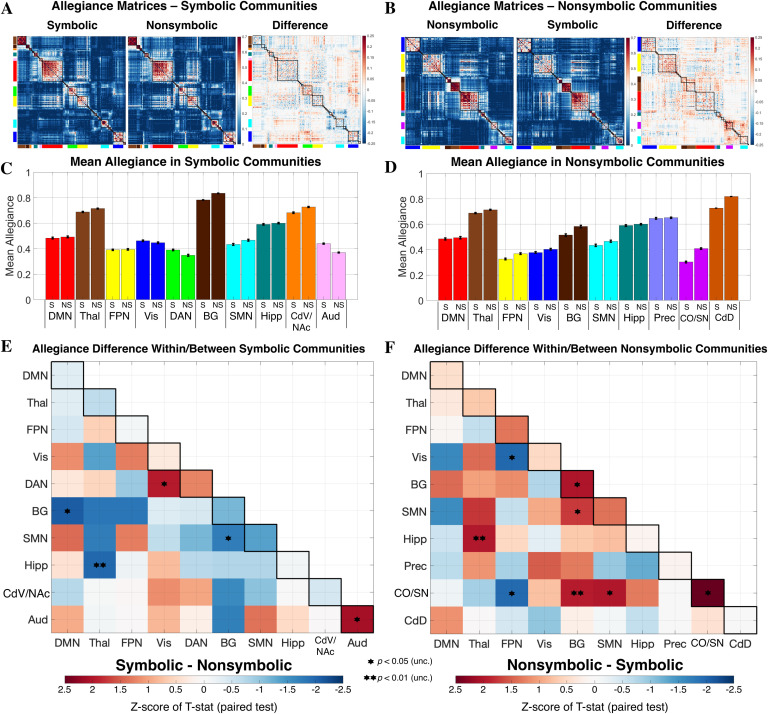
Comparison of community allegiance between formats. (A and B) Group-level allegiance matrices are presented, indicating at each cell in the matrix the proportion of subjects in which two regions were assigned to the same community. Matrix cells are reordered based on the community composition of the symbolic (A) and nonsymbolic (B) formats, respectively, but the values are identical within each format. (C and D) Mean allegiance across region pairs is depicted for each format and was computed using communities as defined for symbolic and nonsymbolic, respectively. Black bars represent standard error of the mean across region pairs. (E and F) Allegiance difference within and between communities detected in each format. No *p* values survived correction for multiple comparisons (*p* < 0.05, FDR corrected for 55 tests along + below the diagonal). The CdD community was composed of two regions (right/left dorsal caudate) and thus contained only one region-to-region connection. In this case, since a paired *t* test was not possible, the *z* score for CdD represents that of the simple difference between formats. DMN = default mode network; Thal = thalamus; FPN = fronto-parietal network; Vis = visual; DAN = dorsal attention network; BG = basal ganglia; SMN = sensorimotor network; Hipp = hippocampus; CdV/NAc = ventral caudate/nucleus accumbens; Aud = auditory; Prec = precuneus; CO/SN = cingulo-opercular/salience network; CdD = dorsal caudate.

While the previous analysis delineated the functional communities present within each condition separately, the detection of a community is driven in part by the organization of the rest of the network. As such, the existence of a community in one condition but not the other does not necessarily indicate that allegiance among these regions (i.e., within-community allegiance) is stronger in that condition per se. Furthermore, even if community assignments are similar between conditions, differences may exist in the hierarchical organization of the network and/or the degree of integration across communities, properties that can be captured by the strength of between-community allegiance. Allegiance values within or between communities provide an indication of how strongly connected, or “allegiant,” the members of a given community are to each other across subjects. A mean allegiance of 0.8 within a community (Figure 3C and D), for example, specifies that the regions of this community were members of the same community in 80% of participants (on average across region pairs). To quantitatively assess whether statistically significant differences existed at the community level, we extracted the vector of region-to-region connections (allegiance values below the diagonal) from the two group-level matrices (Figure 3A and B), for each pair of communities identified in the symbolic and nonsymbolic conditions. To assess the difference between formats, a paired *t* test was then performed on the vectors of allegiance values (e.g., allegiance across regions of the symbolic DAN community in the symbolic matrix versus the same regions in the nonsymbolic matrix). A Monte Carlo permutation procedure with 50,000 iterations was used to determine the significance of the observed *t* statistics, whereby subject-level partitions from each condition were randomly relabeled, null group-level allegiance matrices were constructed, and allegiance values were compared for each community, constructing a null distribution for each *t* statistic of interest. This represents a nonparametric approach to significance testing that is appropriate for repeated measures (Nichols & Holmes, 2004). *Z* scores for the true (i.e., observed) *t* statistics were computed as (*T*_*true*_ − *μ*_*T*_*null*__)/*σ*_*T*_*null*__, and *p* values were assigned based on the position of the value *t* statistic within the null distribution (Figure 3E and F). No community pairs survived FDR correction for multiple comparison across the 55 tests of within- and between-community allegiance conducted for each set of communities (Figure 3E and F) (Benjamini & Hochberg, 1995). However, given the data-driven, exploratory approach employed here assessing all pairwise associations, we did not expect effect sizes to be strong enough to pass such a stringent criterion. The goal here was rather to look for underlying patterns in the data that could then generate hypotheses for future research. In line with recommendations for exploratory research, we report the outcome of our multiple comparison correction procedure but also present the observed effect sizes (*z* scores) as well as the uncorrected *p* values, with a strong caution that these findings must be confirmed in future studies (Althouse, 2016; Feise, 2002; Rothman, 1990; Streiner, 2015; Thompson, Wright, & Bissett, 2020).

Several differences were observed between formats in regards to within- and/or between-community allegiance based on an uncorrected threshold of *p* < 0.05 (two-tailed) (Figure 3E and F). Of the communities identified in the symbolic condition, stronger allegiance was found for symbolic versus nonsymbolic among regions of the auditory (Aud) community (Aud: *z* score = 2.11, *p* = 0.019). This suggests that not only was an auditory community identified in the symbolic condition and not the nonsymbolic condition, but that the regions of this community are significantly more functionally coupled to each other during symbolic trials. Furthermore, allegiance between the dorsal attention (DAN) and visual (Vis) communities was stronger in the symbolic condition (DAN–Vis: *z* score = 1.97, *p* = 0.015). Weaker allegiance was observed in the symbolic condition between basal ganglia (BG) and both the default mode (DMN) and sensorimotor (SMN) communities (BG–DMN: *z* score = −2.09, *p* = 0.010; BG–SMN: *z* score = −1.70, *p* = 0.016), and between the hippocampal (Hipp) and thalamic (Thal) communities (Hipp–Thal: *z* score = −1.97, *p* = 0.004). Of the communities identified in the nonsymbolic condition, stronger allegiance (nonsymbolic > symbolic) was found among regions of BG and cingulo-opercular/salience network (CO/SN) community (BG: *z* score = 2.02, *p* = 0.021; CO/SN: *z* score = 2.50, *p* = 0.005). In nonsymbolic condition, the CO/SN and BG communities were also more allegiant to each other (CO/SN–BG: *z* score = 1.94, *p* = 0.004), and both more allegiant to the SMN community (CO/SN–SMN: *z* score = 2.00, *p* = 0.014; BG–SMN: *z* score = 1.73, *p* = 0.016). Additionally, greater allegiance between the Hipp and Thal communities was observed in the nonsymbolic condition (Hipp–Thal: *z* score = 1.98, *p* = 0.004). Weaker allegiance in the nonsymbolic condition was observed between the FPN and both the Vis and and CO/SN communities (FPN–Vis: *z* score = −1.98, *p* = 0.022; FPN–CO/SN: *z* score = − 1.96, *p* = 0.021).

We additionally asked whether the differences between formats we observed in community allegiance could have been captured by a simpler analysis of raw connectivity strengths. We therefore assessed the relation between allegiance and connectivity values for each format separately, and then critically, whether connectivity information provided a similar pattern of statistically significant differences between formats. These analyses were performed using the median group-level connectivity matrices, group-level allegiance matrices, and the communities as defined at *γ* = 2.45. We first constructed two 10 × 10 matrices representing the mean allegiance and mean connectivity within/between the 10 communities, for each format separately. We then computed the correlation between these matrices, excluding the mirrored values above the diagonal. In other words, we asked, “before taking the difference between formats, are community-level allegiance and connectivity values related?” We found that (as expected) these values were highly related (Pearson *r* = 0.64, *p* < 0.001 in the symbolic data; Pearson *r* = 0.55, *p* < 0.001 in the nonsymbolic data). Next, to determine whether a similar pattern of statistically significant differences could be observed using the connectivity data, we correlated the values depicted in the matrices of Figure 3E and F with those in Supporting Information Figure S3 (excluding values above the diagonal). Neither set of results were significantly correlated (Pearson *r* = 0.07, *p* = 0.60 using symbolic communities; Pearson *r* = 0.23, *p* = 0.08 using nonsymbolic communities), though a marginal positive effect was observed using the nonsymbolic communities. We found the effect sizes of the values within these matrices (i.e., *z* scores of difference between formats, determined via our Monte Carlo procedure) were relatively smaller in the connectivity data (Supporting Information Figure S3) compared with the allegiance data (Figure 3E and F). This analysis highlights the divergence between connectivity and allegiance metrics, and suggests that an allegiance-based approach is sensitive to changes in community topology that are not captured by simple connectivity differences.

### Region-level Allegiance Profiles Across Topological Scales

To provide insights into how the functional roles of particular regions may differ based on number format, we investigated the extent to which node-level allegiance profiles changed between the symbolic and nonsymbolic conditions. In the community-level analysis, we used modularity maximization to define communities of interest and followed this up with quantitative analysis of allegiance differences between formats. This process necessitated a particular setting of the resolution parameter (*γ* = 2.45). Regions, on the other hand, make up the fundamental units of the network and were predetermined by the atlas parcellation, so region-level analyses need not be constrained to a particular topological scale. We reasoned that a more comprehensive assessment of regional allegiance profiles would be to summarize differences between formats across a large range of resolutions (Betzel & Bassett, 2016). We thus performed the following analysis of allegiance profile (dis)similarity for every resolution step across our range of interest, *γ* = 0.05–5, with steps of 0.05 (see Methods for more details on this range).

**Figure 4.**
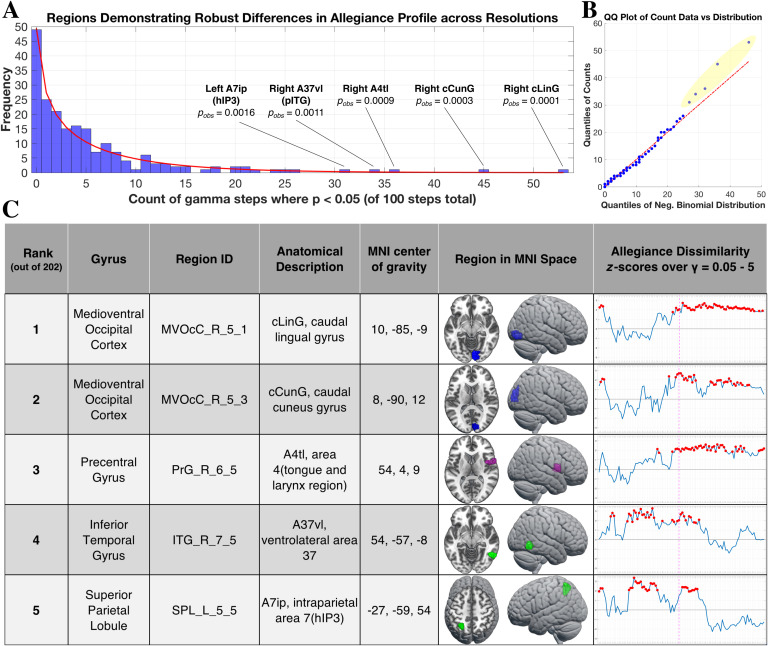
Regions showing robust differences in allegiance profile between formats. (A) Histogram plot showing counts of significant steps over the resolution sweep from all 202 regions, in terms of a significant dissimilarity in allegiance profile between the symbolic and nonsymbolic conditions (*p* < 0.05, uncorrected). The red line indicates the fitted negative binomial distribution, and *p*_*obs*_ indicates the probability of the selected count, given the distribution of all counts observed. (B) Quantile-quantile (QQ) of the fitted distribution (*x*-axis) with respect to the sample distribution (*y*-axis). Note the five regions highlighted in yellow that show considerable deviation from what was expected given the counts demonstrated by the rest of the regions. (C) Table of the five regions showing the most robust differences in allegiance, with *z* scores over the resolution sweep plotted in the rightmost column. The dashed magenta line indicates *γ* = 2.45, the setting for the community-level analysis (Figure 3) and the red dot indicates *p* < 0.05 at that step. Both occipital regions were members of the visual community in the symbolic and nonsymbolic conditions (at *γ* = 2.45), so are presented as blue. The precentral gyrus region was a part of the CO/SN community (magenta) in the nonsymbolic community (and not selected in the symbolic condition). The pITG and hIP3 regions were part of the DAN community in the symbolic condition (green). In the nonsymbolic condition, the hIP3 region was part of the FPN community while the pITG region was not selected (see Supporting Information Table S1 for the community assignments of all regions).

The allegiance profile of a region was defined as the vector of group-level allegiance values from one region to all others, which characterizes a region’s position in the network in relation to the network’s community structure. We computed the (dis)similarity of a region’s allegiance profile between the symbolic and nonsymbolic conditions using Pearson correlation coefficient converted to Fisher Z values, with lower values indicating greater dissimilarity. The significance of dissimilarity was assessed using a similar Monte Carlo permutation approach as before, involving 10,000 random reshufflings of the subject-level partitions to construct a null distribution of group-level allegiance matrices (Nichols & Holmes, 2004). From these null matrices, a *z* score and *p* value for the observed dissimilarity coefficient (inverse of Fisher Z) was computed for every region. To characterize the overall robustness of allegiance profile differences across the resolution sweep, we counted the number of steps in which a region demonstrated a significant dissimilarity in its allegiance profile between formats (*p* < 0.05 one-tailed, uncorrected), and the counts are depicted as a histogram in Figure 4A. In order to reduce the set of regions to those that showed the greatest total count, we first fit a negative binomial curve to the observed distribution, which is commonly used to model overdispersed count data (Bliss & Fisher, 1953; Hartley, 1958; White & Bennetts, 1996). We then assessed the goodness of fit of this theoretical distribution to the observed counts using a quantile-quantile plot (Figure 4B). In general, there was high overlap in the observed versus fitted distribution, with the exception of five outlying regions (highlighted in yellow, Figure 4B): the (1) right caudal lingual gyrus, (2) right caudal cuneus gyrus, (3) right area 4 in precentral gyrus, (4) right ventrolateral area 37 in pITG, and (5) left intraparietal area 7 (hIP3). These five regions demonstrated higher counts than expected given the observed pattern, indicating that, relative to other regions, they showed robust differences in community allegiance between formats across a wide range of organizational scales. We highlight these regions in Figure 4C, including their anatomical description and location, as well as plots of their dissimilarity *z* scores across the full resolution sweep.

**Figure 5.**
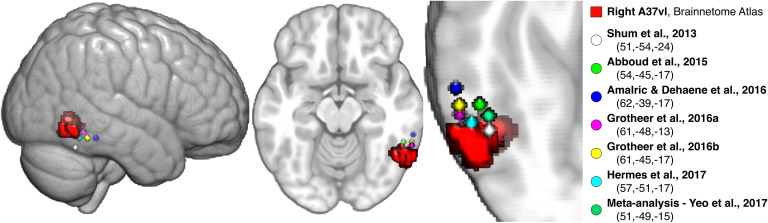
Posterior ITG region (ventrolateral area 37) relative to reported “Number Form Area” coordinates. The ventrolateral area 37 (A37vl) Brainnetome region in the posterior ITG showed significant differences in its allegiance profile between the symbolic and nonsymbolic conditions (see the region-level allegiance analysis in Figure 4). This region is depicted (red) along with spheres centered at the peak MNI coordinates (x, y, z) from several recent studies demonstrating Arabic numeral-selectivity versus other stimuli, using either fMRI or electrocorticography measurements, as well as from an fMRI meta-analysis of numeral versus other symbol processing (Abboud et al., 2015; Amalric & Dehaene, 2016; Fan et al., 2016; Grotheer, Ambrus, et al., 2016; Grotheer, Herrmann, et al., 2016; Hermes et al., 2017; Shum et al., 2013; Yeo et al., 2017). Where coordinates were reported in Talairach space, the tal2mni.m function was used to convert to MNI space via the Brett transform (http://imaging.mrc-cbu.cam.ac.uk/downloads/MNI2tal/tal2mni.m). The A37vl region sits immediately posterior to these coordinates, and was the only ITG region in the Brainnetome Atlas that survived our signal dropout threshold (the others are more anterior).

The right pITG region sits posterior but immediately adjacent to the putative “Number Form Area” as described in recent literature. The volumetric location of this region relative to the peak coordinates reported across multiple studies, including an fMRI meta-analysis, is depicted in Figure 5 (Abboud, Maidenbaum, Dehaene, & Amedi, 2015; Amalric & Dehaene, 2016; Grotheer, Ambrus, et al., 2016; Grotheer, Herrmann, et al., 2016; Hermes et al., 2017; Shum et al., 2013; Yeo et al., 2017). Both the right pITG and left intraparietal hIP3 regions were part of the DAN community—one part of the dual fronto-parietal networks identified in the symbolic condition (Figures 2 and 3; Supporting Information Table S1).

As a supplementary analysis, we investigated the extent to which the observed differences in regional allegiance profiles related to differences in the raw connectivity profiles of each region, using the median group-level connectivity matrices in place of the group-level allegiance matrices (Supporting Information Figure S4). We found that the count of significant steps over the resolution sweep was positively related to the degree of connectivity dissimilarity (Spearman’s *rho* = 0.202, *p* = 0.004), indicating that, albeit a modest relation, there is overlap between allegiance and connectivity at the region level. However, the specific regions showing the greatest counts versus connectivity dissimilarity differed. For instance, three regions showing a high count from the allegiance analysis (right caudal lingual gyrus, right area 4, and left intraparietal hIP3) demonstrated connectivity dissimilarity *z* scores near 0. Along with Supporting Information Figure S3, this indicates that allegiance and connectivity information are largely independent and that allegiance-based analyses can detect changes in regional community membership regardless of strong and/or consistent changes in the region’s raw connectivity profile.

## DISCUSSION

Little is currently known regarding the network architecture of brain systems supporting numerical cognition. While region-based analyses have provided important insights into the neural mechanisms of numerical cognition, a growing body of work outside the numerical cognition field suggests that understanding the network topology of those mechanisms, as well as how this topology is reconfigured across task states, can provide additional meaningful insights (e.g., Braun et al., 2015; Cohen & D’Esposito, 2016; Cole, Bassett, Power, Braver, & Petersen, 2014; Hearne, Cocchi, Zalesky, & Mattingley, 2017; Shine et al., 2019). In this study, we used measurements of task-evoked functional connectivity and network-based analysis to assess the similarities and differences between symbolic and nonsymbolic numerical magnitude processing. Our findings provide evidence for a similar global topology across formats, but selective differences in community and region-level organization.

### No Difference in Global Modularity Between Formats

In regards to functional connectivity networks, the modularity index has been considered as a measure of segregated versus integrated information processing among functional subsystems, with lower modularity indicating greater integration across the brain (Bertolero, Yeo, Bassett, & D’Esposito, 2018; Godwin, Barry, & Marois, 2015; Shine et al., 2016). Our results showed no evidence of a difference in global modularity in subject-level connectivity networks at any topological scale (Figure 1), suggesting that the balance of segregated versus integrated information processing among subnetworks of the brain is similar between formats. It is worth noting, however, that the task employed here involved comparison of each stimulus to the reference number 5, with symbolic and nonsymbolic trials intermixed across the experiment. We speculate that global topological differences between formats may be more pronounced when more than one stimulus must be processed, as is the case in tasks using simultaneous or sequential presentation of two stimuli to be compared, and/or if comparison tasks are performed separately for each format. For instance, in separate tasks, global topological differences may emerge from the adoption of different strategies or attentional states (Bertolero et al., 2018; Cocchi et al., 2014; Cohen & D’Esposito, 2016; Heinzle, Wenzel, & Haynes, 2012), rather than a difference in symbolic versus nonsymbolic processing per se. Our use of intermixed stimuli prevented participants from anticipating one format versus another, thus our results are likely to reflect format-dependent processing differences, rather than strategic differences related to task context.

### Community Allegiance Versus Functional Connectivity

The remaining analyses focused on a comparison of network architectures between number formats at the subnetwork (i.e., community) and region levels. Our approach leveraged the concept of community allegiance, which characterizes region-to-region associations in terms of shared membership among functional subnetworks (see Results section for a more thorough introduction). Intuitively, functional connectivity indexes the extent to which two regions directly interact, whereas allegiance indexes the tendency for two regions to be associated with the same community. Compared with connectivity, allegiance-based analysis is in principle more robust to noise and intersubject variance, and has been shown to be particularly sensitive to reconfigurations in brain network architectures (Bassett et al., 2015). We found that while connectivity and allegiance measures were related (which is expected, given that allegiance is derived from partitions of connectivity matrices), they also diverged considerably (see Results, as well as Supporting Information Figures S3 and S4). The present study indicates that an allegiance-based approach offers a unique and complimentary perspective on network organization, and suggests that future research assessing functional brain networks may benefit from the methodology described herein.

### Fronto-parietal Unification and Fractionation Between Formats

In contrast to the global level, our community-level results revealed that the topography of fronto-parietal control systems differed considerably between formats. Specifically, during nonsymbolic trials, the fronto-parietal network (FPN) was a unitary community composed of 33 regions, while during symbolic trials, these regions were largely fractionated into two distinct communities, a dorsal attention (DAN) community and more ventrally/anteriorly positioned FPN (Figure 2A and B). Distinctions have previously been made between dorsal and ventral attention systems (Corbetta & Shulman, 2002). Previous literature suggests the DAN is involved in the voluntary deployment of attentional control based on goals and expectations as well as ultimately linking stimuli to responses, whereas the ventral network is involved in reorienting of attention to relevant stimuli (Corbetta, Patel, & Shulman, 2008; Ptak, Schnider, & Fellrath, 2017). Through top-down signaling, the DAN in particular is thought to bias sensory areas to be responsive to appropriate features of inputs, and is often found to be functionally connected to visual areas in the ventral occipitotemporal (vOT) cortex (in line with the inclusion of the right pITG region in the symbolic DAN community in the current data; see further discussion below and Figure 2A) (Corbetta et al., 2008; Fox, Corbetta, Snyder, Vincent, & Raichle, 2006; Ptak, 2012; A. C. Vogel, Miezin, Petersen, & Schlaggar, 2012). While both formats may engage the DAN, we speculate that nonsymbolic dot arrays place greater demand on the (ventral) attentional reorienting system by containing multiple objects organized randomly across space. While we did vary the location of the digits across trials (e.g., see Figure 2), it may be that the perceptual regularity of the symbolic stimuli leads to reduced interactions between the DAN and more ventral/anterior components of the FPN during task performance (Vossel, Geng, & Fink, 2014). A potentially related finding came from a recent study by Dixon et al. (2018), who showed that an FPN subsystem (“FPCN_B_”), which has a qualitatively similar spatial profile to our symbolic FPN community, was integrated with the DAN across a range of task states involving externally directed attention and perception. The other component of the FPN, the “FPCN_A_,” was more integrated with the default mode network (DMN) and recruited during internally directed thought (Dixon et al., 2018). The authors suggest that the FPCN_B_ represents task-relevant information (e.g., rules and response mappings) and exerts moment-to-moment, top-down control of the DAN, allowing the DAN to deploy this information in the guidance of spatial attention (e.g., saccades) and ultimately action initiation (Baldauf & Desimone, 2014; Bichot, Heard, DeGennaro, & Desimone, 2015; Dixon et al., 2018). The unified FPN/DAN community we observed in the nonsymbolic condition supports this account, possibly reflecting a stronger engagement of this distributed, visuospatial attentional control system when processing dot sets compared to single digits.

It is important to note that while Power et al. (2011) and Yeo et al. (2011) distinguish separate dorsal attention, ventral attention, and fronto-parietal networks, their boundaries (along with the cingulo-opercular network; Dosenbach, Fair, Cohen, Schlaggar, & Petersen, 2008) are not completely consistent across the literature, nor with our own data-driven community assignments. During task performance, multiple components of these networks are recruited and likely integrated to form a so-called “task-positive” network (Dwyer et al., 2014; Fox et al., 2005). The extent to which each of these putative systems separately contribute to cognitive and attentional control is an ongoing area of research (Petersen & Posner, 2012). In general, our findings indicate that symbolic and nonsymbolic comparison differentially engage large-scale association networks, and further, suggest that assessment of community-level topology may help to incorporate canonical mechanisms of cognitive control and attention into our understanding of number processing.

### Cingulo-Opercular/Salience Network and Basal Ganglia Allegiance Increases During Nonsymbolic Processing

A second pattern of results at the community level involved stronger allegiance, that is, shared community membership, within and between the cingulo-opercular/salience (CO/SN) and basal ganglia (BG) communities in the nonsymbolic condition (Figure 3F). The CO network has been implicated in tonic alertness and task-set maintenance, and demonstrates sustained activity across trials of a cognitive task (Dosenbach et al., 2007; Sadaghiani & D’Esposito, 2015). As part of the dual control network architecture proposed by Dosenbach et al. (2008), the CO operates in parallel to the FPN, which is instead thought to be involved in phasic alertness and adaptive control on a trial-level basis. Our beta-series approach specifically focused on coupling in trial-level responses, and thus is expected to be less sensitive to coupling extending across multiple trials. And even so, the same “task-set” should have been engaged regardless of format, given the trials were intermixed and the same comparison operation was required. Thus, it is difficult to interpret the difference we observed between formats involving CO/SN allegiance within the context of the dual control network framework. On the other hand, this community was a hybrid between the CO and SN communities in the Power et al. (2011) parcellation. The SN is involved in the detection of relevant stimuli, including both internal and external cues, and, for instance, is particularly engaged during oddball paradigms in response to surprising or deviant cues (Menon, 2015). It is implicated in a broad range of goal-directed cognitive functions and is thought to integrate processing among disparate functional subnetworks, potentially filtering input to the ventral attention system (Chang, Yarkoni, Khaw, & Sanfey, 2013; Corbetta et al., 2008). The increased allegiance during nonsymbolic comparison within the CO/SN community in our data may be due to the fact that dot arrays simply involve more visual objects to detect and process, requiring stronger communication among regions in this network. However, this interpretation is highly speculative and further empirical investigation is clearly required to elucidate the mechanisms underlying this finding.

We also observed significantly increased allegiance among regions of the basal ganglia (BG) community, including bilateral putamen, globus pallidus, ventral caudate, and nucleus accumbens (Figure 2B) in the nonsymbolic versus symbolic condition (Figure 3F). BG structures are generally implicated in motor initiation and control, including eye movements, but are also known to participate in multiple cognitive processes such as inhibition, working memory, decision-making, and learning (Helie, Chakravarthy, & Moustafa, 2013; Leisman, Braun-Benjamin, & Melillo, 2014; Middleton & Strick, 2000; Packard & Knowlton, 2002; Wei & Wang, 2016). Multiple parallel cortico-basal ganglia loops are thought to be required during a stimulus-response task, where some cognitive operation is performed (e.g., < or >5) and then linked to a motor output (e.g., press left or right) (Guthrie, Leblois, Garenne, & Boraud, 2013). Since symbolic and nonsymbolic trials required the same motor responses, our finding of increased integration among BG for nonsymbolic trials may relate to the longer response times in the nonsymbolic condition (Yarkoni, Barch, Gray, Conturo, & Braver, 2009). Interestingly, BG regions are often shown to coactivate with the SN, and our observation of greater BG–CO/SN allegiance in the nonsymbolic condition (Figure 3F) further suggests a mechanistic link between these findings (Menon, 2015). Given the diverse roles of the BG, CO, and SN systems in cognition, however, the pattern of increased allegiance among these communities is difficult to interpret and requires future investigation.

### Symbolic Processing Engages an Auditory Community

A final result of interest from the community-level analysis was the observation of the auditory (Aud) community for symbolic trials (Figures 2 and3E). The Aud community was left lateralized and extended along the left superior and middle temporal gyri (STG/MTG; i.e., Brodmann area 22), involving a set of auditory cortex regions involved in the representation of phonemes and phonological retrieval (Binder, 2015; Liebenthal, Binder, Spitzer, Possing, & Medler, 2005). Left temporal regions overlapping with the Aud community found here have been both theoretically (Dehaene & Cohen, 1997) and empirically (Prado, Mutreja, & Booth, 2014) implicated in verbal arithmetic fact retrieval, but their role in symbolic quantity processing has been less well appreciated. The comparison task we employed here does not explicitly require a digit to be mapped to its phonological representation, and thus we did not predict a priori the engagement of a superior temporal network. However, our analysis indicates this system forms a coherent functional community during only the symbolic condition, potentially indicating the implicit binding of number symbols to their spoken word forms. These results are in line with previous studies showing increased functional activation in the superior/middle temporal cortex during symbolic compared with nonsymbolic number comparison (Holloway et al., 2010; Castaldi, Vignaud, & Eger, 2019) and addition (Van Der Ven, Takashima, Segers, Fernández, & Verhoeven, 2016). Our analysis suggests a functional network involving bilateral superior temporal cortex regions is engaged during symbolic but not nonsymbolic number comparison.

### Robust Differences in Community Allegiance of Visual Regions Between Formats

In our region-level analysis we asked, at each step across the resolution range, whether a region showed a difference between formats in its profile of community membership. To identify those regions that showed a robust effect (i.e., regardless of topological scale), we counted the number of steps in which a significant difference was observed. The two regions showing the highest count were located in the right ventromedial occipital cortex (Figure 4). While we controlled for total occupied area and luminance across formats, it is possible that the visual processing streams engaged by the symbolic and nonsymbolic stimuli quickly diverge, and that this is reflected in differential patterns of community affiliation for primary visual cortex as well as higher level visual regions, specifically observed here in the caudal cuneus and lingual gyri, respectively. In a study of symbolic and nonsymbolic processing by Holloway et al. (2010), a univariate contrast between formats found multiple occipital areas more engaged by nonsymbolic stimuli, and this was after subtracting out activation associated with control conditions that were matched in terms of total pixels. Our findings of allegiance differences involving visual regions seem convergent, and could reflect engagement of object location, individuation, and summation mechanisms thought to underlie nonsymbolic number processing (Verguts & Fias, 2004). Indeed, these stages are proposed to serve as the inputs necessary for numerical magnitude representation in parietal cortex, and have been shown to involve an occipitoparietal pathway including the inferior and middle occipital gyri (Roggeman, Santens, Fias, & Verguts, 2011). Symbolic numerals may take an alternative route to their associated magnitude representations, bypassing the multi-object normalization and accumulation mechanisms required to process dot sets (Bulthé et al., 2019; Santens, Roggeman, Fias, & Verguts, 2010), and instead engage a ventral stream object-recognition pathway extending into the ITG (Hannagan, Amedi, Cohen, Dehaene-Lambertz, & Dehaene, 2015; Harel, 2016; Price & Devlin, 2011). We hesitate to make any mechanistic interpretations, but our data at least indicate that the “position” of these visual regions with respect to community topology is significantly altered between formats. Taken together with the trend of greater allegiance during symbolic trials between the Visual and DAN as well as FPN communities (Figure 3E and F), our results build on prior univariate work, suggesting that number format influences not only local activity but also the distributed interactions of occipital regions.

### Robust Differences in Community Allegiance of the pITG and hIP3 Between Formats

A growing body of evidence suggests that number symbol processing engages a region in posterior inferior temporal gyrus (pITG) that preferentially responds to digits compared to other symbols (Grotheer, Herrmann, et al., 2016; Hermes et al., 2017; Pollack & Price, 2019; Shum et al., 2013). Given this literature, we hypothesized that if this region is preferentially engaged during the recognition of Arabic numerals, it would show a greater tendency to join a functional community involving parietal areas during the symbolic trials. Among the 202 regions analyzed here, the right pITG (Figure 5) was one of only five regions demonstrating robust differences in its allegiance profile between conditions (Figure 4). Its affiliation with the symbolic DAN community (Figure 2 and Supporting Information Table S1) provides evidence that interactions between the right pITG and superior parietal cortex and intraparietal sulcus (IPS) are particularly relevant for processing number symbols. This is in line with recent electrocorticography and fMRI work showing coupling between lateral vOT and parietal areas during numeral recognition and arithmetic tasks, including behavioral relevance of task-evoked coupling for math ability and developmental increases over adolescence (Battista et al., 2018; Daitch et al., 2016). Even at rest a similar pattern of robust connectivity is observed, specifically involving pITG to IPS regions (Abboud et al., 2015; Nemmi, Schel, & Klingberg, 2018). Building on this literature, we found that connectivity patterns of the pITG are differentiated in a task context, and that selective coupling of the pITG with parietal regions during symbolic processing occurs even while performing a simple magnitude comparison task. Our analysis further indicates that the topological position of the pITG with respect to large-scale community organization is a distinguishing feature between formats, suggesting the pITG plays a pivotal role in the symbolic number processing network. The pITG to parietal coupling we observed here may reflect a mechanism by which visual number forms are “mapped” to magnitude representations in the IPS. Alternatively, though not mutually exclusive, this interaction may represent a more general process of recurrent exchange between neural populations in the vOT (tuned to learned sets of visual features) and the DAN (involved in feature-based, attentional control), whereby presentation of a preferred stimulus (e.g., a number symbol) elicits efficient attentional capture, allowing for identification and further task-level processing. Such an argument has been made for the role of the visual word form area in reading and why it shows similarly strong connectivity to the DAN (A. C. Vogel et al., 2012; note the visual word form area is near the pITG region described here, albeit in left vOT). Whatever the case, the present results, together with other recent findings, suggest work looking into the mechanistic contributions of the pITG and its functional interactions will be a fruitful avenue for future research in numerical cognition.

Finally, we found that a region in the hIP3 subdivision of the left IPS demonstrated robust differences in its allegiance profile between formats. The IPS has been extensively implicated in number processing tasks and thought to be involved in the representation of both symbolic and nonsymbolic magnitude information (Ashkenazi, Henik, Ifergane, & Shelef, 2008; Dehaene et al., 2003; Fias, Lammertyn, Reynvoet, Dupont, & Orban, 2003; Piazza et al., 2004). Compared with the right IPS, the left IPS has been shown to be more sharply tuned to Arabic numerals than to nonsymbolic arrays (Piazza, Pinel, Le Bihan, & Dehaene, 2007) and is also thought to be more affected by the ontogenetic process of learning number symbols (Ansari, 2008; Cantlon et al., 2006; Rivera, Reiss, Eckert, & Menon, 2005; S. E. Vogel, Goffin, & Ansari, 2015). Interestingly, Santens et al. (2010) reported a differential connectivity profile of the left IPS between symbolic and nonsymbolic processing, despite this region being equally activated by both formats. Our findings are convergent, demonstrating that the left hIP3 takes on a distinct pattern of community membership between formats, and that this distinction is pronounced relative to other brain regions. This line of evidence indicates that, while both formats may engage the left IPS, the functional pathways to and from this region are divergent between symbolic and nonsymbolic processing, and highlights the additional level of insight afforded by connectivity-based approaches. It is important to note that the analysis we have performed here does not directly speak to the question of whether symbolic and nonsymbolic number engage shared representations in the IPS, as has been a topic of intense study in recent years (e.g., Bulthé, De Smedt, & Op de Beeck, 2015, 2018; Lyons, Ansari, & Beilock, 2015). Our results instead provide complementary evidence that the whole-brain functional architectures involving left hIP3 are different between formats, and suggest that a more comprehensive understanding of hIP3 function during number processing will require a characterization of this region’s role with respect to functional network dynamics. Furthermore, given previous reports that symbolic processing in the left IPS changes over development, longitudinal studies looking at the trajectory of this region’s connectivity profile over learning may provide mechanistic insights into the acquisition of symbolic number abilities.

### Limitations

One limitation of this work is that our community-level analyses were conducted at a particular setting of the structural resolution parameter (*γ* = 2.45), which influences the size and composition of the detected communities (e.g., see Methods) (Fortunato & Hric, 2016). We determined this setting based on the heuristic of maximizing the balance across regions of community stability and change between conditions (Mattar et al., 2015). The goal here was to be sensitive to differences in community organization while avoiding an unrealistic scenario in which a majority of regions change their community affiliation (i.e., assuming that there is some common functional architecture between formats). For other questions, such as “What is the shared community structure across task states?,” using a heuristic where partitions are maximally consistent between conditions may be more appropriate. At other settings of *γ*, the number of communities as well as regional allegiance profiles will change (e.g., see Methods), and thus additional patterns of community-level differences between formats may have been observed.

Another limitation to note is that the Brainnetome Atlas we used to define our nodes represents a parcellation of the brain at particular spatial scale (*n* = 246 regions) and was derived based on structural-connectivity patterns. We chose this atlas because it has been shown to capture areas with relatively homogenous functional connectivity profiles (Fan et al., 2016). However, node definitions at different spatial scales or based on different properties, for example, anatomical, architectonic, or random subdivisions, may demonstrate different patterns from what we observed (Fornito, Zalesky, & Breakspear, 2013; Sporns, 2014; Zalesky et al., 2010).

### Conclusions

In summary, in this study we adopted a data-driven, network neuroscience approach to characterize the whole-brain functional architecture supporting symbolic and nonsymbolic number comparison. Our findings provide several novel insights. A unified fronto-parietal control network in the nonsymbolic condition was fractionated into FPN and DAN communities during symbolic number processing. We found evidence for stronger allegiance among a left-lateralized Auditory network in the symbolic condition, potentially reflecting access to phonological representations of number word forms. The right pITG, proximal to the putative “Number Form Area,” joined the DAN system in the symbolic condition only, supporting its proposed role in the processing of number symbols. Both the right pITG and the hIP3 subdivision of the left IPS demonstrated robust differences in their community allegiance profiles between formats. Taken together, these results reveal a pattern of overlapping and distinct network architectures for symbolic and nonsymbolic number processing, and highlight the additional level of insight provided by analysis of functional network topology.

## METHODS

### Participants

Forty neurologically healthy, right-handed subjects participated in the study for undergraduate course credit. Of those recruited, 6 participants were excluded from analyses due to high levels of motion and 1 was excluded for significant signal dropout (exclusion criteria detailed below), resulting in a final sample of 33 participants (19.5 ± 0.9 years, 20 females). All subjects had normal or corrected-to-normal vision. Informed consent was obtained from each subject in accordance with the Vanderbilt University Institutional Review Board policy.

### fMRI Tasks

Participants completed two consecutive runs of an event-related number comparison paradigm. For each trial, participants judged whether an Arabic digit (symbolic) or dot array (nonsymbolic) was less or more than five by pressing a button with either their right index or right middle finger, respectively, as quickly and accurately as possible. A total of 160 trials were presented, composed of 80 symbolic trials and 80 nonsymbolic trials that were intermixed and pseudo-randomly ordered (i.e., no more than 3 consecutive trials were of the same number or format). Stimulus numerosity was either 2, 4, 6, or 8, which occurred at equal probability and was counterbalanced across formats. Stimuli were created using the MATLAB package first described by Piazza et al. (2004). Nonsymbolic stimuli were controlled for total surface area across numerosities by reducing dot size with increasing numerosity. Additionally, stimuli were controlled for total occupied area and luminance across formats (i.e., on average, dot sets contained the same number of pixels as numerals) in an effort to control for nonnumerical visual parameters across trials. Stimulus duration was 500 ms and interstimulus intervals (ISI) ranged from 3,300–7,300 ms, in 1,000-ms increments, with an average of 5,300 ms. ISI was counterbalanced across numerosities and formats.

### Imaging

Imaging was performed using a 7 Tesla (7T) Philips Achieva scanner with a 32-channel head coil. An MP2RAGE (Marques et al., 2010) image was acquired for anatomical reference, aligned to the anterior/posterior commissures, with the following parameters: TR = 4.315 ms, TE = 1.92 ms, flip angle = 7,240 coronal slices, voxel size = 1 mm^3^, imaging matrix = 240 × 240 × 192, acquisition time = 1,010 s. These images were corrected for B1-field inhomogeneities, as well as proton density and T2* effects according to the procedure described by Marques et al. (2010). For the event-related experiment, functional T2*-weighted images were acquired over two runs of 243 volumes each, with the following parameters: TR = 2,000 ms, TE = 25 ms, flip angle = 63, 46 axial slices (with no interslice gap), voxel size = 2.5 mm^3^, imaging matrix = 96 × 96 × 46, acquisition time = 500 s per run (16 m 40 s of functional data total).

### Preprocessing

fMRI data was preprocessed in AFNI (Cox, 1996) using the afni_proc.py program. Preprocessing involved slice-time and motion correction, coregistration, normalization to the MNI152 2009c template using affine registration, smoothing with a 4-mm kernel, and scaling. All transformations of the raw functional data were concatenated and applied in one step to reduce multiple resampling and interpolation errors. Head-motion/outlier censoring was performed by removing volumes from the activation model (described below) that demonstrated between-volume movement of >0.3-mm Euclidean norm distance or if >5% of voxels in a volume (within a brain mask) were determined to be outliers. Across our final sample, the percentage of censored volumes was 4.0 ± 4.1%.

### Beta-Series Estimation

To assess task-related functional connectivity, we used a beta-series correlation method (BSC) (Rissman et al., 2004). This method allows for estimation of condition-specific, task-evoked connectivity by looking at correlated fluctuations in coactivity patterns across multiple stimulus events. Compared with an alternative, commonly used methodology of standard or generalized psychophysiological interaction analysis (PPI/gPPI) (Friston et al., 1997; McLaren, Ries, Xu, & Johnson, 2012), BSC has been shown to be more powerful for detecting task-evoked connectivity in event-related designs that feature many trials, short stimulus durations, and short ISIs, such as we have implemented here (Cisler, Bush, & Steele, 2014). To compute trial-wise beta (i.e., activation) maps, we employed the “least-squares separate” (LS-S) method proposed by Mumford et al. (2012) using AFNI’s 3dLSS function (Geib, Stanley, Dennis, Woldorff, & Cabeza, 2017; Mumford, Turner, Ashby, & Poldrack, 2012). Compared with the original implementation of BSC in which each trial is included as a separate regressor in a single general linear model (GLM), the LS-S method includes a regressor for the trial of interest and then a nuisance regressor with all other trials of interest combined. This method is more computationally efficient and, importantly, was found to produce more accurate estimates of trial-specific activations in simulations (Mumford Turner, Ashby, & Poldrack, 2012). Also included in these subject-level GLMs were the following nuisance regressors: 6 motion parameters, 6 motion derivatives, 0th- to 4th-order Legendre polynomials to model low-frequency drifts (per run), the first five principal components in white matter voxels, and first five principal components in the lateral ventricles. Tissue masks were created using the Computational Anatomy Toolbox (CAT12) in SPM12 (https://www.fil.ion.ucl.ac.uk/spm/software/spm12/) (Gaser & Dahnke, 2016). The ventricle region of interest (ROI) was created by masking the cerebrospinal fluid segmentation with the lateral ventricle regions from the Neuromorphometric Atlas included in CAT12. The inclusion of these tissue-based principal component regressors, known as the aCompCor method, was intended to account for nonneural physiological fluctuations as well as residual motion-related noise and has been shown to improve connectivity estimates in resting-state studies (Caballero-Gaudes & Reynolds, 2017; Ciric et al., 2017; Muschelli et al., 2014). The preprocessed 4D functional data (after smoothing and scaling) served as the inputs into the 3dLSS function for beta-series estimation. Censoring was performed at this stage and resulted in GLMs with a mean of 933 ± 32 volumes (i.e., observations) out of 972 collected volumes. These models included trials/volumes from a separate identification task (not analyzed here) performed at the beginning of the scanning session. The inclusion of all acquired data into one model ensured more accurate estimates of nuisance effects. For each trial a separate GLM was constructed that included a total of 42 nuisance regressors plus a regressor for the trial of interest and one for the sum of all other trial responses. The output of this process involved 3D beta maps for every trial indicating the model coefficient at each voxel, serving as trial-level activation maps that could then be sorted by formats.

### Within-Format Normalization

To ensure that differences in voxel-wise activity levels and/or variance between formats did not confound our estimates of connectivity, prior to extraction of average beta series from ROIs, we implemented a within-format normalization procedure in which the beta maps were first separated by format and concatenated, then each voxel-wise beta series was normalized by subtracting the voxel-wise mean and dividing by the voxel-wise standard deviation. This procedure was adapted from the multivariate pattern analysis literature, in which normalization of beta values is commonly employed prior to classifier training (Misaki, Kim, Bandettini, & Kriegeskorte, 2010). With this procedure, we could be more certain our connectivity estimates were derived from covariance in the *pattern* of trial-level activation, rather than biased by the *magnitude* of activations, per se.

### Beta-Series Extraction and Scrubbing

**Figure 6.**
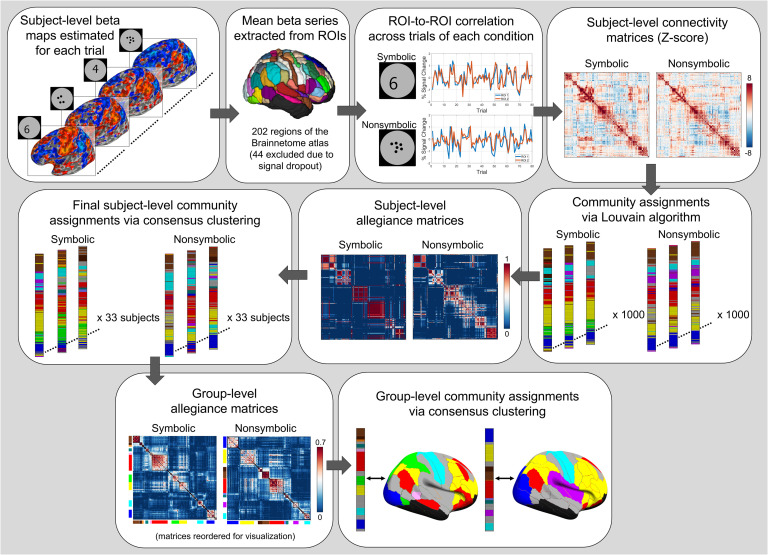
Functional connectivity and consensus clustering pipeline. The data processing pipeline is depicted, starting from trial-level beta map estimation to final group-level allegiance matrices and community assignments. Gray arrows indicate the stepwise progression from one stage of processing to the next. All plots portray representative data from the analysis.

We extracted the average beta series in each of 202 cortical and subcortical ROIs from the Brainnetome Atlas (Fan et al., 2016), which represented the reduced set of ROIs (originally 246) after accounting for signal dropout (Figure 6; see Supporting Information Methods). We chose the Brainnetome Atlas as it a connectivity-based parcellation which provides a biologically plausible set of fine-grained regions in both cortex and subcortex (Fan et al., 2016). Since censoring (i.e., exclusion of high-motion/outlier volumes) was performed in subject-level GLMs, some trials’ convolved response contained fewer volumes in the final model. Note that the hemodynamic response to a single trial lasted approximately 13.1 s and spanned either six or seven volumes of data at our 2-s sampling rate (TR). We expected less reliable beta estimates for trials in which the response profile contained censored volumes. Therefore, we implemented a “beta-scrubbing” approach similar to Ray et al. (2017), in which we excluded trials during which two or more volumes were censored (i.e., the associated beta was removed from the series before running correlations). Across the original sample of 40 subjects, the median number of retained trials was 147 (91.9%). We determined six subjects were outliers in the number of retained trials based on a threshold of 3.5 × the median absolute deviation (MAD), a metric for robust outlier detection (Leys, Ley, Klein, Bernard, & Licata, 2013). These subjects thus demonstrated a significant degree of motion compared with the rest of the sample and were excluded from further analyses, along with one additional subject who demonstrated significant signal dropout (see Supporting Information Methods). In the final sample of 33 subjects, out of 80 trials presented for each format, there was an average of 74.2 ± 5.8 (92.8 ± 7.3%) trials used for symbolic and 74.6 ± 5.2 (93.2 ± 6.4%) used for nonsymbolic. A paired *t* test indicated the number of retained trials was not significantly different between formats (*t*(32) = −1.08, *p* = 0.29).

### Functional Connectivity

To construct subject-level connectivity matrices, we computed the Pearson correlation between all region pairs by using the average beta-series vector from each region, yielding a 202 × 202 matrix that was symmetric about the diagonal (Figure 6). Matrices were constructed for symbolic and nonsymbolic trials separately. Correlation coefficients were Fisher *Z* transformed and converted to *z* scores via dividing by the standard error ($\frac{1}{\sqrt{N-3}}$), where *N* is the number of observations (i.e., betas) retained after the scrubbing procedure (Fisher, 1915). The *z*-score matrices served as the final subject-level matrices for network analyses.

### Modularity Maximization and Consensus Clustering

To characterize network topology during the symbolic and nonsymbolic conditions, we employed the graph-theoretical approach of modularity maximization. This data-driven clustering procedure attempts to partition a network into nonoverlapping communities where each node is assigned to exactly one community, such that there is high within-community and low between-community connectivity (Newman, 2006). The modularity quality function quantifies the degree to which this is achieved given a partition of matrix *w*. Specifically, we employed a generalized Louvain algorithm (Blondel, Guillaume, Lambiotte, & Lefebvre, 2008) that maximized a variant of the standard Newman–Girvan quality function, termed Q*, which incorporates an asymmetric treatment of negative weights, as proposed by Rubinov and Sporns (2011; Dwyer et al., 2014). This modified treatment of negative weights is recommended for functional networks because of differing neurobiological significance of positive and negative correlations in BOLD timeseries (for further discussion, see Rubinov & Sporns, 2011) and is calculated as follows:

$$Q^{*}=\frac{1}{v^{+}}\sum_{ij}(w_{ij}^{+}-\gamma e_{ij}^{+})\delta_{M_{i}M_{j}}-\frac{1}{v^{+}+v^{-}}\sum_{ij}(w_{ij}^{-}-\gamma e_{ij}^{-})\delta_{M_{i}M_{j}}$$

where each brain region *i* is assigned to community *M*_*i*_ and region *j* is assigned to community *M*_*j*_; *δ*_*M*_*i*_*M*_*j*__ = 1 if *M*_*i*_ = *M*_*j*_ and equals 0 otherwise; $e_{ij}^{\pm }$ is the within-community connection strength expected by chance for either positive or negative weights; *v*^+^ and *v*^−^ denote the sum of all positive and negative weights in the network, respectively; and, *γ* is the structural resolution parameter, which influences the size of the detected communities (Reichardt & Bornholdt, 2006) (see below for details of a data-driven approach to choosing a value for this parameter).

The Louvain algorithm uses a two-step procedure in which first every node is assigned to its own community, and iterative moves are made assigning a node to its neighboring community until a maximum Q* is reached. In a second step, the resulting communities are combined into a smaller network that sums the node weights within each community, and this simplified network gets submitted back to the first step for a second pass, and so on, until no changes can be made in step 1 to improve Q* (Blondel et al., 2008). Critically, the resulting community structure is dependent on the random order of moves considered at each pass. In principle, there is some “optimal” partition of the network, that is, a partition or set of partitions exists that returns the highest possible Q* for that matrix. In practice, the optimal partition of a given network is unknown and multiple iterations of modularity maximization result in distinct, though often similar, solutions, referred to as the so-called degeneracy of modular solutions (Good, De Montjoye, & Clauset, 2010). Degeneracy can be leveraged through the use of consensus clustering methods, which aggregate across many partitions of the same network to define a consensus partition (Lancichinetti & Fortunato, 2012). We implemented a multistep approach that involved modularity maximization and consensus clustering at the subject level, then consensus clustering at the group level (Figure 6).

Each fully weighted, subject-level matrix was first partitioned using the generalized community Louvain algorithm (Blondel et al., 2008) (via the community_louvain.m function of the Brain Connectivity Toolbox (BCT), http://www.brain-connectivity-toolbox.net, with the “negative_asym” flag). The algorithm was run 10^3^ times, with each iteration resulting in a single partition of the 202 regions into communities (i.e., a vector of values indicating the community assignment for each region). From these partitions, a 202 × 202 allegiance matrix was constructed which represents, at each cell, the percentage of iterations in which two regions were assigned to the same community (Bassett et al., 2015) (via the agreement_weighted.m function of the BCT). The subject-level allegiance matrix was then further clustered using a consensus clustering procedure to obtain a final, consensus partition of regional communities (Lancichinetti & Fortunato, 2012) (via the consensus_und.m function of the BCT; note the “tau” parameter was set to 0.5 for all analyses herein, which thresholds the allegiance matrix to remove elements weaker than 0.5, i.e., when there is less than 50% agreement across partitions). The final subject-level partitions were then used to create a group-level allegiance matrix that represented, at each cell, the percentage of subjects in which two regions were assigned to the same community. The same consensus clustering procedure was then used to determine the final group-level community assignments (Figure 6). Because assigned community labels are arbitrary, labels were maximally matched between the final partitions of the symbolic and nonsymbolic conditions based on community overlap (via the pair_labeling.m function of the Network Community Toolbox, http://commdetect.weebly.com). As a final step, permutation testing was performed to reduce the set of communities to only those demonstrating significance at the subject level (see Supporting Information Methods).

### Defining the Structural Resolution Parameter

**Figure 7.**
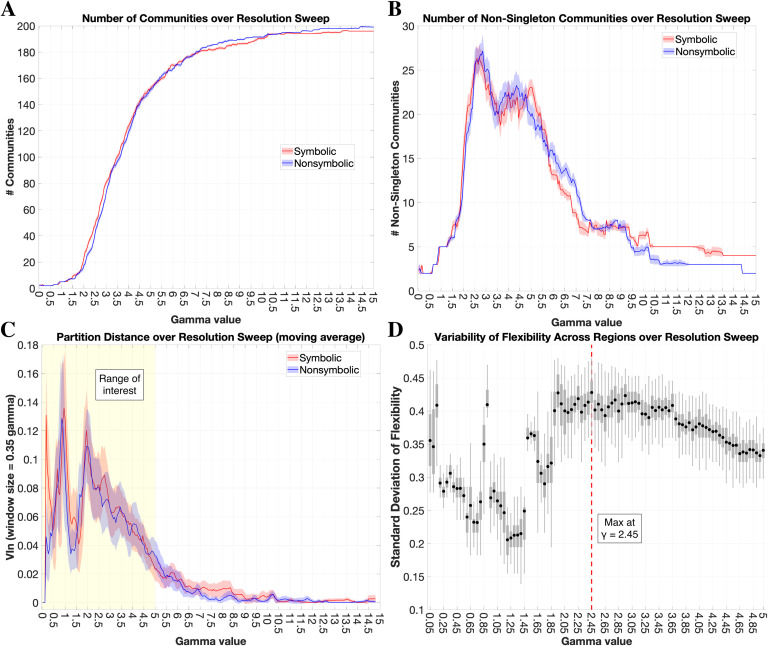
Resolution parameter sweep. (A and B) Results of a parameter “sweep,” in which the structural resolution parameter (*γ*) was varied from 0 to 15 in increments of 0.05, with 100 iterations of the multistep consensus clustering approach at each level of *γ*. The mean for each format is plotted in red and blue, with shaded regions indicating the SD across iterations. (C) Mean partition distance over sliding window of ±3 steps (window size = 0.35 *γ*) is plotted for each format, measured using the normalized variation of information (Meilǎ, 2007). This plot indexes the change in community structure over incremental steps in *γ*, highlighting the point in parameter space in which no new scale of community organization is discovered, at *γ* ≈ 5. We used this result to define the range of interest for our analyses of format differences across resolutions (i.e., *γ* = 0.05–5). (D) The red dashed line indicates the *γ* at which the variability (SD) of flexibility was maximal, with flexibility defined as the number of regions that changed their community assignment between the symbolic and nonsymbolic conditions. The maximum SD would result from half of all regions changing community assignments and the other half maintaining their assignment. The distribution at each gamma value represents the result of all pairwise comparisons of the 100 group-level partitions generated for each format at that step. The mean of this distribution (black circle) was used to determine the optimal gamma setting for further analyses (i.e., *γ* = 2.45).

When applying modularity maximization, an important consideration is the definition of the free parameter, *γ*, referred to as the structural resolution parameter. The effect of *γ* is such that lower values result in fewer and larger communities, and higher values result in more and smaller communities, which provides some constraint on the topological scale of the resulting partitions. We fully sampled this parameter space in Figure 7A and B, varying *γ* from 0.05 to 15. At the highest settings, the algorithm discovered as many communities as there were regions (i.e., all singletons, which refers to regions that are assigned a community unto themselves). To further refine this space to a range of interest, we determined the point at which there was relatively little change in community structure. We used a sliding window approach in which we calculated average normalized variation of information, a measure of partition distance, over community partitions within a window size of 0.35 (Meilǎ, 2007). This procedure revealed that at approximately *γ* = 5, there was little change in community structure, suggesting that a stable set of strongly connected (nonsingleton) communities existed at this topological scale, and that beyond this point, nodes from these communities progressively became singletons and no new community organization was discovered (Figure 7B and C). We therefore adopted the range from *γ* = 0.05 to 5 as our resolution range of interest for all subsequent analyses.

With the range of interest defined, we still needed to determine the ideal setting of *γ* for the community-level analysis (note that for the global- and region-level analyses, we present the results across the full range of interest; Figures 1 and 4). Though setting *γ* to an arbitrary value of 1 is common practice, this is not necessarily optimal. A more principled approach involves “sweeping” across parameter space and using some heuristic to determine the optimal value, for example, where community definitions are most consistent across repeated iterations (Betzel & Bassett, 2016; Betzel et al., 2017; Gu et al., 2015). Since we were interested here in a comparison of network architecture between the symbolic and nonsymbolic conditions, we wanted to conduct our investigation at a resolution that would be sensitive to both similarities *and* differences between formats. To achieve this, we employed a heuristic, used previously by Mattar et al. (2015), where the optimal *γ* value is that which maximizes variability in the flexibility coefficient across regions. Flexibility is defined simply as the proportion of (task) conditions in which a region changes its assigned community (calculated via the flexibility.m function of the Network Community Toolbox). With only two conditions, a region’s flexibility can be 1 or 0, and the *variability* of flexibility is the standard deviation of the vector of 1’s and 0’s (one value for each of the 202 region). By maximizing the variability of flexibility across brain regions, we are looking for a *γ* at which there is a balance between stable and changing community assignments between conditions (i.e., is maximal if exactly half of regions change their assignment between conditions). Importantly, the procedure does not ensure the differences are significant within subjects (which we assess via permutation testing), just that *some* differences and *some* similarities exist between the final group-level partitions of each condition. This approach is thus orthogonal to the prospect of finding reliable within-subject effects. To determine the optimal *γ* setting for further investigation, we performed the multistep group partitioning procedure 100 times at each value of *γ*, ranging from 0.05 to 5 in increments of 0.05 (for this analysis, subject-level matrices were partitioned 100 times with community_louvain.m). For each iteration, we calculated the flexibility of nodal assignments between formats in the final group-level partitions (Figure 7D). At low levels of *γ* there are fewer communities (Figure 7A) and the region assignments are stable between formats, thus resulting in many regions with a flexibility of 0, that is, low variability across regions. At high levels of *γ*, there are many communities, including an increasing number of singletons. After *γ* values ≈ 2.8, the number of nonsingleton communities detected drops, due to the fact that a few previously detected communities break apart into singleton communities. Ultimately, we found a maximum in the mean variability (SD) of flexibility at an intermediate value of *γ* = 2.45. The subsequent community-level analyses involved modularity maximization using this value.

## ACKNOWLEDGMENTS

We thank all the volunteers who participated in this study.

## SUPPORTING INFORMATION

Supporting Information for this article is available at https://doi.org/10.1162/netn_a_00144. Data and code for this project have been made publicly available at https://osf.io/sb5v2/ and https://github.com/conradbn/CR7T_Connectivity, respectively (Conrad et al., 2020a, 2020b).

## AUTHOR CONTRIBUTIONS

Benjamin N. Conrad: Conceptualization; Data curation; Formal analysis; Methodology; Software; Visualization; Writing - Original Draft; Writing - Review & Editing. Eric D. Wilkey: Conceptualization; Data curation; Funding acquisition; Investigation; Methodology; Project administration; Resources; Writing - Review & Editing. Darren J. Yeo: Conceptualization; Data curation; Investigation; Methodology; Writing - Review & Editing. Gavin R. Price: Conceptualization; Data curation; Funding acquisition; Methodology; Project administration; Resources; Supervision; Writing - Review & Editing.

## FUNDING INFORMATION

Gavin R. Price, Peabody College Small Research Grant. Gavin R. Price, National Science Foundation (NSF) Division of Research on Learning in Formal and Informal Settings (http://dx.doi.org/10.13039/100000173), Award ID: 1660816. Gavin R. Price, National Science Foundation (NSF) Division of Research on Learning in Formal and Informal Settings (http://dx.doi.org/10.13039/100000173), Award ID: 1750213. Eric D. Wilkey is the recipient of a BrainsCAN Postdoctoral Fellowship at Western University, funded by the Canada First Research Excellence Fund (CFREF) and Banting Postdoctoral Fellowship funded by the Natural Sciences and Engineering Research Council (NSERC) of Canada. Darren J. Yeo is supported by the Humanities, Arts, and Social Sciences International PhD Scholarship, co-funded by Nanyang Technological University and the Government of Singapore: Ministry of Education.

## TECHNICAL TERMS

Symbolic number processing: Operating on numerical information derived from a learned notation system, e.g., Arabic numerals or number words.
Nonsymbolic number processing: Operating on numerical information derived from sets of perceptual elements, e.g., dot arrays or tone sequences.
Beta-series correlation: A method for measuring functional connectivity in event-related fMRI data that assesses how similarly two regions respond to trials of a given condition.
Community: A subnetwork of nodes (e.g., regions) that are more densely connected to each than to other nodes of the network.
Modularity (*Q*): Indexes the quality ofa network partition relative to a null model and, when maximized, summarizes the extent of segregation versus integration among a network’s communities.
Structural resolution parameter (*γ*): A free variable in the modularity quality function that affects the number and size of communities detected.
Allegiance: The extent to which two regions belong to the same community, measured by the probability of shared community membership across multiple partitions.
Network partition: A decomposition of network (i.e., clustering solution) in which each node is assigned to exactly one community.
Modularity maximization: An algorithmic process to determine a partition that maximizes the modularity quality index (*Q*), i.e., the optimal clustering solution.
Consensus clustering: An algorithm for determining a representative network partition from many partitions of the same network or from multiple subjects.
Monte Carlo permutation: A technique used for nonparametric hypothesis testing in which a null distribution is constructed by randomly resampling observed data.
