# Supplementary material for: Network topology of symbolic and nonsymbolic number comparison
Source: Netw Neurosci. 2020 Aug 1;4(3):714–45. doi: 10.1162/netn_a_00144 (PMC7462424; doi:10.1162/netn_a_00144)
Supplement: Supplementary file 1 [file netn-04-714-s001.pdf]

Conrad, B. N., Wilkey, E. D., Yeo, D. J., & Price, G. R. (2020). Supporting information for "Network topology of symbolic and nonsymbolic number comparison." *Network Neuroscience*, *4*(3), 714–745. https://doi.org/10.1162/netn_a_00144

# Supplementary Materials for:

# Network topology of symbolic and nonsymbolic number comparison

Benjamin N. Conrad[1,2], Eric D. Wilkey[1,2,3], Darren J. Yeo[1,2,4], and Gavin R. Price[1,2]

[1]Psychology and Human Development, Vanderbilt University, Nashville, TN, USA

[2]Vanderbilt Brain Institute, Vanderbilt University, Nashville, TN, USA

[3]Brain & Mind Institute, Western University, London, ON, Canada

[4]Division of Psychology, School of Social Sciences, Nanyang Technological University, Singapore

# Contents of this Supplementary Document


1. **Supplementary Methods**
    a. Region Exclusion based on Signal Dropout
    b. Community Selection
2. **Supplementary Results**
    a. Figure S1 - Community Selection
    b. Figure S2 - Dual-Network Architecture
    c. Figure S3 - Median Connectivity in Selected Communities - Comparison Within/Between Formats
    d. Figure S4 - Relationship of Region-level Allegiance Profile Differences to Connectivity Dissimilarity
    e. Supplementary Table S1 - Region table with community assignments
3. **References**

# Supplementary Methods

## Region Exclusion based on Signal Dropout

Signal dropout is common in orbitofrontal and inferior temporal areas in fMRI due to close proximity to air-tissue interfaces. This is problematic because signal attenuation will lead to unreliable activation estimates in affected regions and thus subsequent connectivity measures could be driven by noise. While studies of functional connectivity seldom report whether they have accounted for signal dropout (e.g., through EPI masking), this procedure has recently been discussed as an important step in connectivity analyses (Peer, Abboud, Hertz, Amedi, & Arzy, 2016). To address this, we took a quantitative approach to characterizing signal dropout and determining regions to exclude from whole-brain network analyses. First, we sought to determine the optimal fraction of signal to consider as "usable" via AFNI's 3dAutomask function (i.e., the "clip fraction" parameter). We found the default clip fraction of 0.5 to be sub-optimal, as it excluded relatively more voxels in our 7T EPI data compared to its application in 3T data, due to a much higher range of values across the brain and a heavy-tailed distribution whereby exterior areas of cortex had as much as four times the signal as those located more centrally/inferiorly. We can consider the histogram of voxel-wise signals in EPI images as a mixture of two distributions, one encompassing non-brain/attenuated-signal voxels and another encompassing brain/water-signal voxels. We varied the clip fraction in 3dAutomask across a range of values and looked for the first setting at which the overall group mean distribution showed no local minima (i.e., the surviving voxels can be expected to come from the second, brain/water distribution), arriving at a clip fraction of 0.32. As a second step, we used the resulting EPI masks to determine the percentage of usable voxels in each region for each subject.

We set an exclusion criterion such that every subject had to have at least 50% usable voxels in a region for that region to be included in further analyses. One subject had 41 of 246 regions with less than 50% coverage due to significant dropout artifacts. This was more than 3.5 × the median absolute deviation (MAD), a robust metric for outlier detection, and was excluded from further analyses (median number of regions excluded across subjects = 24 ± 3.5 MAD) (Leys, Ley, Klein, Bernard, & Licata, 2013). We justified a stringent cutoff of 50% coverage based on the idea that if we aim to adequately characterize the function/connectivity of a region, we should do so based on usable signal from *at least* half of the voxels in this region. A similar cutoff has been employed in previous studies (e.g., Geerligs, Rubinov, Cam-CAN, & Henson, 2015). This procedure resulted in a final set of 202 regions out of 246 from the Brainnetome atlas (Fan et al., 2016).

## Community Selection

Using the final group-level partitions, we computed the modularity contribution ($Q_c^*$) of each community, *c*, in the subject-level connectivity matrices, with $Q_c^*$ defined as follows:

$$Q_c^* = \frac{1}{v^+}\sum_{ij\in c}(w_{ij}^+ - \gamma e_{ij}^+) - \frac{1}{v^+ + v^-}\sum_{ij\in c}(w_{ij}^- - \gamma e_{ij}^-)$$

Null distributions were created by reshuffling the partition vector $10^4$ times, preserving the number and size of communities across permutations, and recalculating $Q^*_c$ for each community, in accordance with the procedure performed by Betzel et al. (2017). *Z*-scores were calculated for each true value by subtracting the mean and dividing by the standard deviation across permutations. This was done for each subject, and we selected a community if its modularity contribution exceeded the 99$^{th}$ percentile of the null distribution in more than half of subjects,

with the reasoning that, to be considered for further analyses, the group-level communities must demonstrate significant connectivity at the subject level.

# Supplementary Results

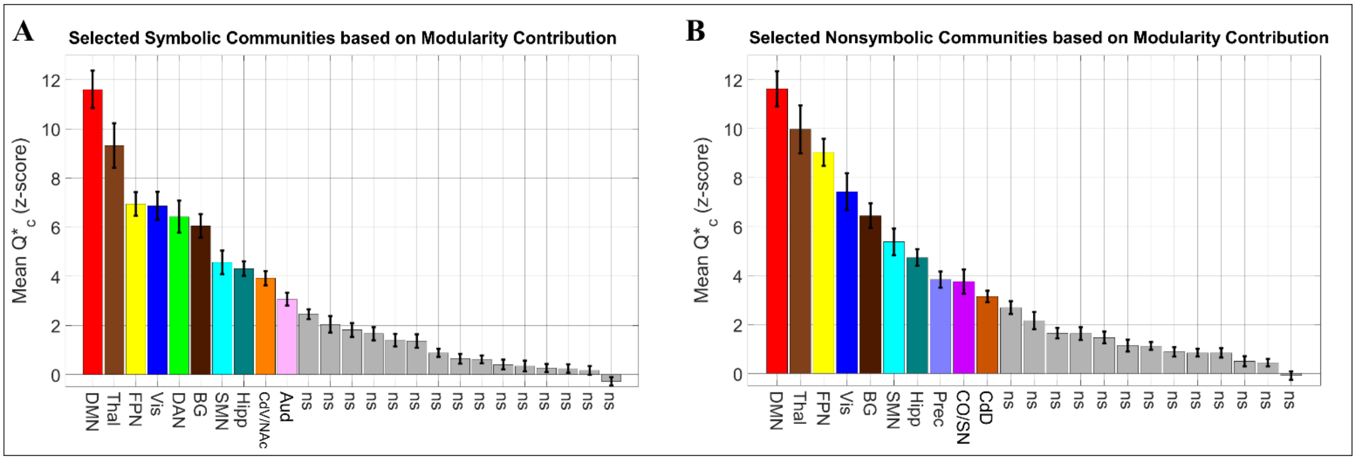


**Supplementary Figure S1 - Community Selection**. Communities were selected based on their modularity contribution ($Q*_c$) in subject-level connectivity matrices. Colored communities indicate that these passed our selection criterion, such that the modularity contribution exceeded the 99$^{th}$ percentile of the null distribution in more than half of subjects, after permutation testing. DMN = Default mode network; Thal = Thalamus; FPN = Frontoparietal network; Vis = Visual; DAN = Dorsal attention network; BG = Basal ganglia; SMN = Sensorimotor network; Hipp = Hippocampus; CdV/NAc = Ventral caudate/Nucleus accumbens; Aud = Auditory; Prec = Precuneus; CO/SN = Cingulo-opercular/Salience network; CdD = Dorsal caudate; ns = not selected.

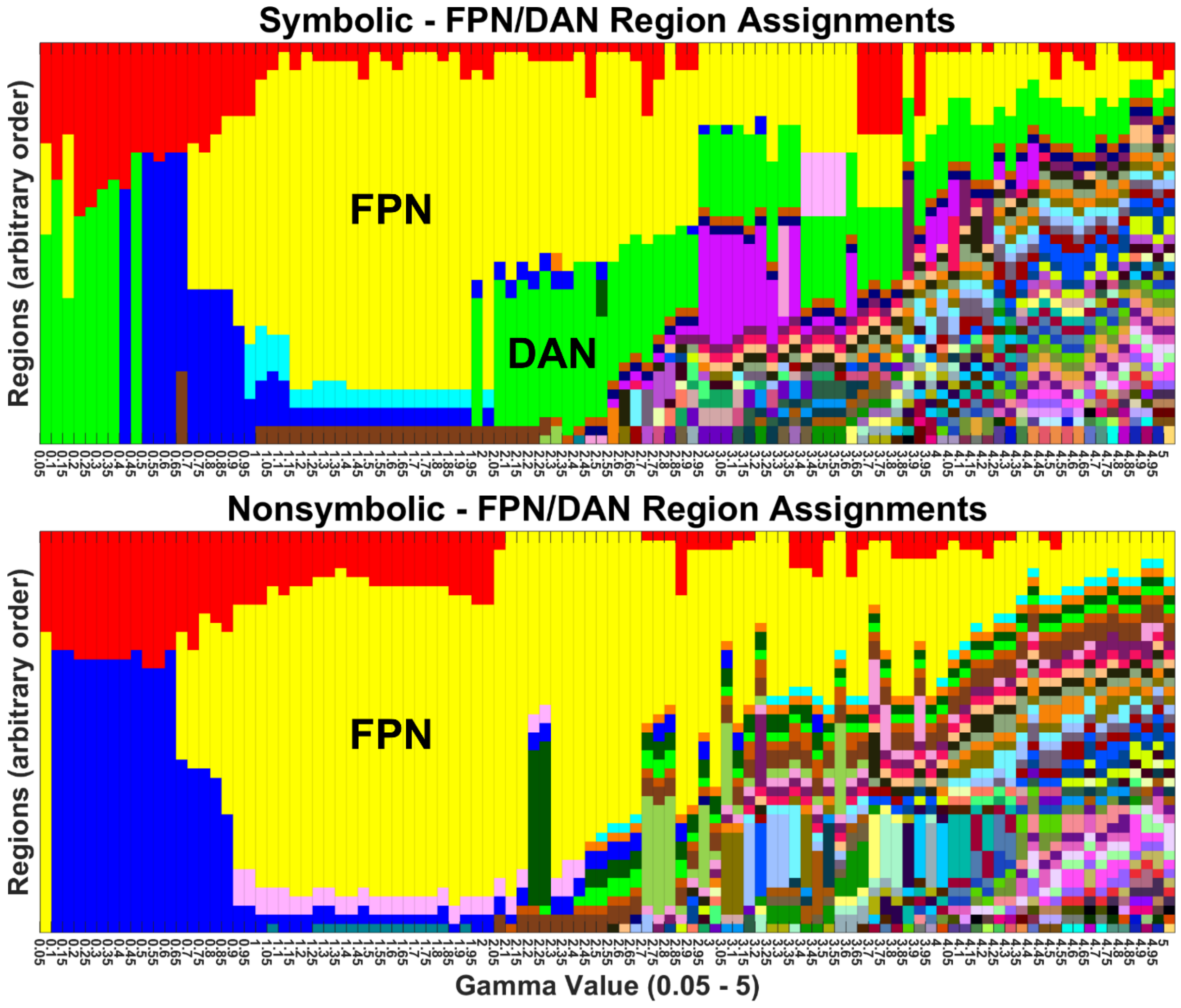


**Supplementary Figure S2 - Dual-Network Architecture.** Dual network architecture of FPN and DAN for symbolic trials is robust across a range of gamma values ($\gamma$ = 1.95 - 2.9) while nonsymbolic shows a consistent single network (FPN) architecture. Regions include the union of all those in the symbolic FPN, symbolic DAN, and nonsymbolic FPN communities defined in Fig. 2A, B. Partitions were relabeled at each step based on their maximal overlap with the communities at $\gamma$ = 2.45 within each format respectively, which was our $\gamma$ setting of interest for the community-level analyses (see Fig. 2 and 3 in the main manuscript). Note that at the low end of the $\gamma$ range, FPN/DAN regions are subsumed by a hierarchical task-positive/task-negative community structure where the task-negative community includes primarily default mode regions (red) and the task-positive community includes visual (blue) and DAN (green) regions.

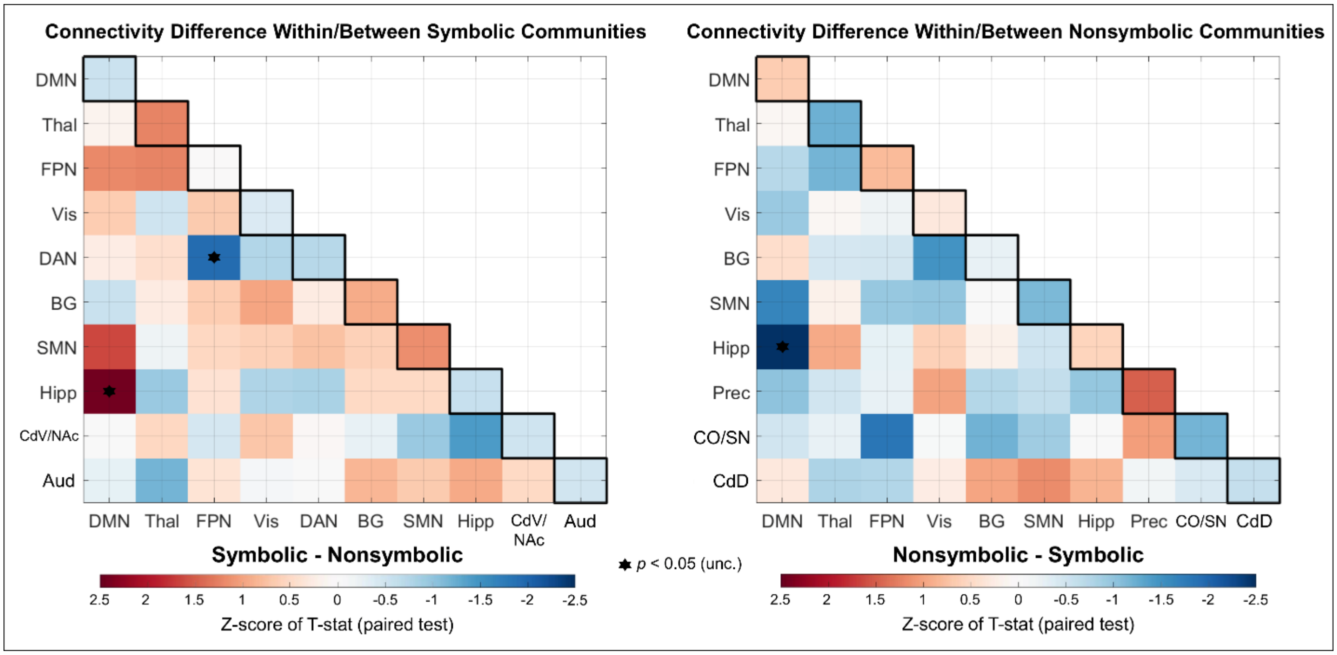


**Supplementary Figure S3 – Median Connectivity in Selected Communities - Comparison Between Formats**. The median region-to-region connectivity values (i.e., median across subject-level correlation z-score matrices) within or between each community were vectorized, and compared between formats using paired *t*-tests. Non-parametric significance was determined using Monte Carlo permutations of the subject-level matrices from each format, with 50,000 iterations to mirror the group-level allegiance analysis presented in Fig. 3E, F. The *z*-score for CdD represents that of the simple difference between formats, since there was only one region-to-region connection within this community and a paired *t*-test was not possible. Uncorrected *p*-values below 0.05 (two-tailed) are indicated with a star. No effects demonstrated an uncorrected *p*-value below 0.01 (two-tailed) and none survived correction for multiple comparisons ($p < 0.05$, FDR correction for 55 tests along + below the diagonal). Compared to the allegiance results in the main manuscript (Fig. 3E, F), the connectivity-based effect sizes were relatively smaller (mean absolute value ± SD = 0.68 ± 0.47 versus 0.88 ± 0.61 using symbolic communities; 0.73 ± 0.50 versus 0.96 ± 0.63 using nonsymbolic communities). Furthermore, the two sets of results

were not significantly correlated (Pearson $r = 0.07$, $p = 0.60$ using symbolic communities; Pearson $r = 0.23$, $p = 0.08$ using nonsymbolic communities), indicating these analyses provide distinct information. Interestingly, the connectivity comparison shows reduced coupling among the DAN and FPN communities in the symbolic condition (DAN-FPN $z$-score = -1.95, $p$ = 0.0246), mirroring the distinction between formats in the dual versus unified DAN/FPN architecture (see Supplementary Fig. S2). DMN = Default mode network; Thal = Thalamus; FPN = Frontoparietal network; Vis = Visual; DAN = Dorsal attention network; BG = Basal ganglia; SMN = Sensorimotor network; Hipp = Hippocampus; CdV/NAc = Ventral caudate/Nucleus accumbens; Aud = Auditory; Prec = Precuneus; CO/SN = Cingulo-opercular/Salience network; CdD = Dorsal caudate.

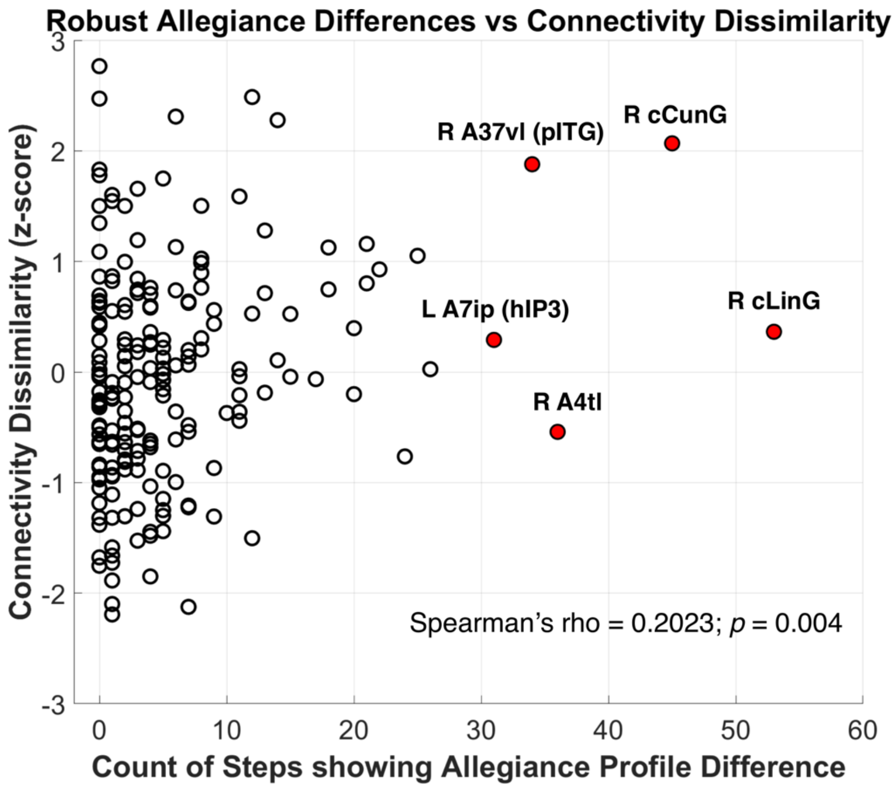


**Supplementary Figure S4 – Relationship of Region-level Allegiance Profile Differences to Connectivity Dissimilarity.** The median region-to-region connectivity matrices (i.e., median across subject-level correlation z-score matrices) were constructed for the symbolic and nonsymbolic condition (same data used in Supplementary Fig. S3). Each region's connectivity profile (pairwise connectivity strength with all other regions) was extracted and correlated between formats. The resulting Fisher Z values provided a measure of (dis)similarity in connectivity profile between formats. The Monte Carlo procedure with 50,000 permutations was performed to derive dissimilarity *z*-scores (higher *z*-score indicates lower Fisher Z compared to chance). Scatter plot shows a small, but significant relationship between these *z*-scores and the total count of steps over the resolution sweep showing a significant difference in allegiance profile between formats (see Fig. 4 in the main manuscript). However, note that the regions showing the strongest differences in connectivity profile (top left of plot) do not show robust

differences in community allegiance. It could be the case, for instance, that a region demonstrates a significant difference between formats in overall connectivity strength, but this region's community membership remains stable. Furthermore, the regions showing the most robust differences in community allegiance (red) (Fig. 4), did not necessarily show a reliable difference in connectivity profiles between formats. This result suggests that while allegiance changes are related to differences in connectivity patterns, these metrics provide distinct, complementary information. R = right; L = left; A37vl = ventrolateral area 7; pITG = posterior inferior temporal gyrus; A7ip = intraparietal area 7; hIP3 = horizontal intraparietal sulcus subdivision 3; A4tl = area 4 (tongue and larynx region); cCunG = caudal cuneus gyrus; cLingG = caudal lingual gyrus.

| LabelID | Lobe | Gyrus | Name | Anatomical and modified Cyto-architectonic descriptions | MNI center of gravity (X,Y,Z) | Community Symbolic | Community Nonsymbolic |
|---|---|---|---|---|---|---|---|
| 1 | Frontal Lobe | SFG, Superior Frontal Gyrus | SFG_L_7_1 | A8m, medial area 8 | -5 ,15, 54 | Unselected | Unselected |
| 2 | | | SFG_R_7_1 | A8m, medial area 8 | 7, 16, 54 | Unselected | Unselected |
| 3 | | | SFG_L_7_2 | A8dl, dorsolateral area 8 | -18, 24, 53 | DMN | DMN |
| 4 | | | SFG_R_7_2 | A8dl, dorsolateral area 8 | 22, 26, 51 | DMN | DMN |
| 5 | | | SFG_L_7_3 | A9l, lateral area 9 | -11, 49, 40 | DMN | DMN |
| 6 | | | SFG_R_7_3 | A9l, lateral area 9 | 13, 48, 40 | DMN | DMN |
| 7 | | | SFG_L_7_4 | A6dl, dorsolateral area 6 | -18, -1, 65 | Unselected | Unselected |
| 8 | | | SFG_R_7_4 | A6dl, dorsolateral area 6 | 20, 4, 64 | Unselected | Unselected |
| 9 | | | SFG_L_7_5 | A6m, medial area 6 | -6, -5, 58 | Unselected | Unselected |
| 10 | | | SFG_R_7_5 | A6m, medial area 6 | 7, -4, 60 | Unselected | Unselected |
| 11 | | | SFG_L_7_6 | A9m,medial area 9 | -5, 36, 38 | DMN | DMN |
| 12 | | | SFG_R_7_6 | A9m,medial area 9 | 6, 38, 35 | DMN | FPN |
| 13 | | | SFG_L_7_7 | A10m, medial area 10 | -8, 56, 15 | DMN | DMN |
| 14 | | | SFG_R_7_7 | A10m, medial area 10 | 8, 58, 13 | DMN | DMN |
| 15 | | MFG, Middle Frontal Gyrus | MFG_L_7_1 | A9/46d, dorsal area 9/46 | -27, 43, 31 | Unselected | Unselected |
| 16 | | | MFG_R_7_1 | A9/46d, dorsal area 9/46 | 30, 37, 36 | Unselected | Unselected |
| 17 | | | MFG_L_7_2 | IFJ, inferior frontal junction | -42, 13, 36 | DMN | DMN |
| 18 | | | MFG_R_7_2 | IFJ, inferior frontal junction | 42, 11, 39 | FPN | FPN |
| 19 | | | MFG_L_7_3 | A46, area 46 | -28, 56, 12 | DMN | DMN |
| 20 | | | MFG_R_7_3 | A46, area 46 | 28, 55, 17 | FPN | FPN |
| 21 | | | MFG_L_7_4 | A9/46v, ventral area 9/46 | -41, 41, 16 | FPN | FPN |
| 22 | | | MFG_R_7_4 | A9/46v, ventral area 9/46 | 42, 44, 14 | FPN | FPN |
| 23 | | | MFG_L_7_5 | A8vl, ventrolateral area 8 | -33, 23, 45 | DMN | DMN |
| 24 | | | MFG_R_7_5 | A8vl, ventrolateral area 8 | 42, 27, 39 | DMN | FPN |
| 25 | | | MFG_L_7_6 | A6vl, ventrolateral area 6 | -32, 4, 55 | Unselected | FPN |
| 26 | | | MFG_R_7_6 | A6vl, ventrolateral area 6 | 34, 8, 54 | FPN | FPN |
| 27 | | | MFG_L_7_7 | A10l, lateral area10 | -26, 60, -6 | Dropout | Dropout |
| 28 | | | MFG_R_7_7 | A10l, lateral area10 | 25, 61, -4 | Dropout | Dropout |
| 29 | | IFG, Inferior Frontal Gyrus | IFG_L_6_1 | A44d,dorsal area 44 | -46, 13, 24 | FPN | Unselected |
| 30 | | | IFG_R_6_1 | A44d,dorsal area 44 | 45, 16, 25 | FPN | FPN |
| 31 | | | IFG_L_6_2 | IFS, inferior frontal sulcus | -47, 32, 14 | DMN | DMN |
| 32 | | | IFG_R_6_2 | IFS, inferior frontal sulcus | 48, 35, 13 | FPN | FPN |
| 33 | | | IFG_L_6_3 | A45c, caudal area 45 | -53, 23, 11 | DMN | DMN |
| 34 | | | IFG_R_6_3 | A45c, caudal area 45 | 54, 24, 12 | DMN | FPN |
| 35 | | | IFG_L_6_4 | A45r, rostral area 45 | -49, 36, -3 | DMN | DMN |
| 36 | | | IFG_R_6_4 | A45r, rostral area 45 | 51, 36, -1 | DMN | FPN |
| 37 | | | IFG_L_6_5 | A44op, opercular area 44 | -39, 23, 4 | FPN | FPN |
| 38 | | | IFG_R_6_5 | A44op, opercular area 44 | 42, 22, 3 | FPN | FPN |
| 39 | | | IFG_L_6_6 | A44v, ventral area 44 | -52, 13, 6 | FPN | FPN |
| 40 | | | IFG_R_6_6 | A44v, ventral area 44 | 54, 14, 11 | FPN | FPN |
| 41 | | OrG, Orbital Gyrus | OrG_L_6_1 | A14m, medial area 14 | -7, 54, -7 | DMN | DMN |
| 42 | | | OrG_R_6_1 | A14m, medial area 14 | 6, 47, -7 | DMN | DMN |
| 43 | | | OrG_L_6_2 | A12/47o, orbital area 12/47 | -36, 33, -16 | Dropout | Dropout |
| 44 | | | OrG_R_6_2 | A12/47o, orbital area 12/47 | 40, 39, -14 | DMN | DMN |
| 45 | | | OrG_L_6_3 | A11l, lateral area 11 | -23, 38, -18 | Dropout | Dropout |
| 46 | | | OrG_R_6_3 | A11l, lateral area 11 | 23, 36, -18 | Dropout | Dropout |
| 47 | | | OrG_L_6_4 | A11m, medial area 11 | -6, 52, -19 | Dropout | Dropout |
| 48 | | | OrG_R_6_4 | A11m, medial area 11 | 6, 57, -16 | Dropout | Dropout |
| 49 | | | OrG_L_6_5 | A13, area 13 | -10, 18, -19 | Dropout | Dropout |
| 50 | | | OrG_R_6_5 | A13, area 13 | 9, 20, -19 | Dropout | Dropout |
| 51 | | | OrG_L_6_6 | A12/47l, lateral area 12/47 | -41, 32, -9 | DMN | DMN |
| 52 | | | OrG_R_6_6 | A12/47l, lateral area 12/47 | 42, 31, -9 | DMN | FPN |
| 53 | | PrG, Precentral Gyrus | PrG_L_6_1 | A4hf, area 4(head and face region) | -49, -8, 39 | SMN | SMN |
| 54 | | | PrG_R_6_1 | A4hf, area 4(head and face region) | 55, -2, 33 | SMN | SMN |
| 55 | | | PrG_L_6_2 | A6cdl, caudal dorsolateral area 6 | -32, -9, 58 | Unselected | Unselected |
| 56 | | | PrG_R_6_2 | A6cdl, caudal dorsolateral area 6 | 33, -7, 57 | Unselected | Unselected |
| 57 | | | PrG_L_6_3 | A4ul, area 4(upper limb region) | -26, -25, 63 | SMN | SMN |
| 58 | | | PrG_R_6_3 | A4ul, area 4(upper limb region) | 34, -19, 59 | SMN | SMN |
| 59 | | | PrG_L_6_4 | A4t, area 4(trunk region) | -13, -20, 73 | SMN | SMN |
| 60 | | | PrG_R_6_4 | A4t, area 4(trunk region) | 15, -22, 71 | SMN | SMN |
| 61 | | | PrG_L_6_5 | A4tl, area 4(tongue and larynx region) | -52, 0, 8 | Unselected | CO/SN |
| 62 | | | PrG_R_6_5 | A4tl, area 4(tongue and larynx region) | 54, 4, 9 | Unselected | CO/SN |
| 63 | | | PrG_L_6_6 | A6cvl, caudal ventrolateral area 6 | -49, 5, 30 | FPN | FPN |
| 64 | | | PrG_R_6_6 | A6cvl, caudal ventrolateral area 6 | 51, 7, 30 | Unselected | FPN |
| 65 | | PCL, Paracentral Lobule | PCL_L_2_1 | A1/2/3ll, area1/2/3 (lower limb region) | -8, -38, 58 | SMN | SMN |
| 66 | | | PCL_R_2_1 | A1/2/3ll, area1/2/3 (lower limb region) | 10, -34, 54 | SMN | SMN |
| 67 | | | PCL_L_2_2 | A4ll, area 4, (lower limb region) | -4, -23, 61 | SMN | SMN |
| 68 | | | PCL_R_2_2 | A4ll, area 4, (lower limb region) | 5, -21, 61 | SMN | SMN |
| 69 | | STG, Superior Temporal Gyrus | STG_L_6_1 | A38m, medial area 38 | -32, 14, -34 | Dropout | Dropout |
| 70 | | | STG_R_6_1 | A38m, medial area 38 | 31, 15, -34 | Dropout | Dropout |
| 71 | | | STG_L_6_2 | A41/42, area 41/42 | -54, -32, 12 | Unselected | Unselected |
| 72 | | | STG_R_6_2 | A41/42, area 41/42 | 54, -24, 11 | Unselected | Unselected |
| 73 | | | STG_L_6_3 | TE1.0 and TE1.2 | -50, -11, 1 | Unselected | Unselected |
| 74 | | | STG_R_6_3 | TE1.0 and TE1.2 | 51, -4, -1 | Unselected | Unselected |
| 75 | | | STG_L_6_4 | A22c, caudal area 22 | -62, -33, 7 | Aud | DMN |
| 76 | | | STG_R_6_4 | A22c, caudal area 22 | 66, -20, 6 | Unselected | Unselected |
| 77 | | | STG_L_6_5 | A38l, lateral area 38 | -45, 11, -20 | Unselected | Unselected |
| 78 | | | STG_R_6_5 | A38l, lateral area 38 | 47, 12, -20 | Unselected | Unselected |
| 79 | | | STG_L_6_6 | A22r, rostral area 22 | -55, -3, -10 | Aud | Unselected |
| 80 | | | STG_R_6_6 | A22r, rostral area 22 | 56, -12, -5 | Unselected | Unselected |

| LabelID | Lobe | Gyrus | Name | Anatomical and modified Cyto-architectonic descriptions | MNI center of gravity (X,Y,Z) | Community Symbolic | Community Nonsymbolic |
|---|---|---|---|---|---|---|---|
| 81 | Temporal Lobe | MTG, Middle Temporal Gyrus | MTG_L_4_1 | A21c, caudal area 21 | -65, -30, -12 | Dropout | Dropout |
| 82 | | | MTG_R_4_1 | A21c, caudal area 21 | 65, -29, -13 | Dropout | Dropout |
| 83 | | | MTG_L_4_2 | A21r, rostral area 21 | -53, 2, -30 | Dropout | Dropout |
| 84 | | | MTG_R_4_2 | A21r, rostral area 21 | 51, 6, -32 | Dropout | Dropout |
| 85 | | | MTG_L_4_3 | A37dl, dorsolateral area37 | -59, -58, 4 | Unselected | Unselected |
| 86 | | | MTG_R_4_3 | A37dl, dorsolateral area37 | 60, -53, 3 | Unselected | Unselected |
| 87 | | | MTG_L_4_4 | aSTS, anterior superior temporal sulcus | -58, -20, -9 | DMN | DMN |
| 88 | | | MTG_R_4_4 | aSTS, anterior superior temporal sulcus | 58, -16, -10 | DMN | Unselected |
| 89 | | ITG, Inferior Temporal Gyrus | ITG_L_7_1 | A20iv, intermediate ventral area 20 | -45, -26, -27 | Dropout | Dropout |
| 90 | | | ITG_R_7_1 | A20iv, intermediate ventral area 20 | 46, -14, -33 | Dropout | Dropout |
| 91 | | | ITG_L_7_2 | A37elv, extreme lateroventral area37 | -51, -57, -15 | Dropout | Dropout |
| 92 | | | ITG_R_7_2 | A37elv, extreme lateroventral area37 | 53, -52, -18 | Dropout | Dropout |
| 93 | | | ITG_L_7_3 | A20r, rostral area 20 | -43, -2, -41 | Dropout | Dropout |
| 94 | | | ITG_R_7_3 | A20r, rostral area 20 | 40, 0, -43 | Dropout | Dropout |
| 95 | | | ITG_L_7_4 | A20il, intermediate lateral area 20 | -56, -16, -28 | Dropout | Dropout |
| 96 | | | ITG_R_7_4 | A20il, intermediate lateral area 20 | 55, -11, -32 | Dropout | Dropout |
| 97 | | | ITG_L_7_5 | A37vl, ventrolateral area 37 | -55, -60, -6 | Unselected | Unselected |
| 98 | | | ITG_R_7_5 | A37vl, ventrolateral area 37 | 54, -57, -8 | DAN | Unselected |
| 99 | | | ITG_L_7_6 | A20cl, caudolateral of area 20 | -59, -42, -16 | Dropout | Dropout |
| 100 | | | ITG_R_7_6 | A20cl, caudolateral of area 20 | 61, -40, -17 | Dropout | Dropout |
| 101 | | | ITG_L_7_7 | A20cv, caudoventral of area 20 | -55, -31, -27 | Dropout | Dropout |
| 102 | | | ITG_R_7_7 | A20cv, caudoventral of area 20 | 54, -31, -26 | Dropout | Dropout |
| 103 | | FuG, Fusiform Gyrus | FuG_L_3_1 | A20rv, rostroventral area 20 | -33, -16, -32 | Dropout | Dropout |
| 104 | | | FuG_R_3_1 | A20rv, rostroventral area 20 | 33, -15, -34 | Dropout | Dropout |
| 105 | | | FuG_L_3_2 | A37mv, medioventral area37 | -31, -64, -14 | Unselected | Vis |
| 106 | | | FuG_R_3_2 | A37mv, medioventral area37 | 31, -62, -14 | Unselected | Vis |
| 107 | | | FuG_L_3_3 | A37lv, lateroventral area37 | -42, -51, -17 | Dropout | Dropout |
| 108 | | | FuG_R_3_3 | A37lv, lateroventral area37 | 43, -49, -19 | Dropout | Dropout |
| 109 | | PhG, Parahippocampal Gyrus | PhG_L_6_1 | A35/36r, rostral area 35/36 | -27, -7, -34 | Dropout | Dropout |
| 110 | | | PhG_R_6_1 | A35/36r, rostral area 35/36 | 28, -8, -33 | Dropout | Dropout |
| 111 | | | PhG_L_6_2 | A35/36c, caudal area 35/36 | -25, -25, -26 | Dropout | Dropout |
| 112 | | | PhG_R_6_2 | A35/36c, caudal area 35/36 | 26, -23, -27 | Dropout | Dropout |
| 113 | | | PhG_L_6_3 | TL, area TL (lateral PPHC, posterior | -28, -32, -18 | Hipp | Hipp |
| 114 | | | PhG_R_6_3 | TL, area TL (lateral PPHC, posterior | 30, -30, -18 | Dropout | Dropout |
| 115 | | | PhG_L_6_4 | A28/34, area 28/34 (EC, entorhinal cortex) | -19, -12, -30 | Dropout | Dropout |
| 116 | | | PhG_R_6_4 | A28/34, area 28/34 (EC, entorhinal cortex) | 19, -10, -30 | Dropout | Dropout |
| 117 | | | PhG_L_6_5 | TI, area TI(temporal agranular insular cortex) | -23, 2, -32 | Dropout | Dropout |
| 118 | | | PhG_R_6_5 | TI, area TI(temporal agranular insular cortex) | 22, 1, -36 | Dropout | Dropout |
| 119 | | | PhG_L_6_6 | TH, area TH (medial PPHC) | -17, -39, -10 | Hipp | Hipp |
| 120 | | | PhG_R_6_6 | TH, area TH (medial PPHC) | 19, -36, -11 | Hipp | Hipp |
| 121 | | pSTS, posterior Superior Temporal Sulcus | pSTS_L_2_1 | rpSTS, rostroposterior superior temporal | -54, -40, 4 | Aud | DMN |
| 122 | | | pSTS_R_2_1 | rpSTS, rostroposterior superior temporal | 53, -37, 3 | Aud | Unselected |
| 123 | | | pSTS_L_2_2 | cpSTS, caudoposterior superior temporal | -52, -50, 11 | Aud | DMN |
| 124 | | | pSTS_R_2_2 | cpSTS, caudoposterior superior temporal sulcus | 57, -40, 12 | Unselected | Unselected |
| 125 | Parietal Lobe | SPL, Superior Parietal Lobule | SPL_L_5_1 | A7r, rostral area 7 | -16, -60, 63 | DAN | Unselected |
| 126 | | | SPL_R_5_1 | A7r, rostral area 7 | 19, -57, 65 | DAN | Unselected |
| 127 | | | SPL_L_5_2 | A7c, caudal area 7 | -15, -71, 52 | DAN | FPN |
| 128 | | | SPL_R_5_2 | A7c, caudal area 7 | 19, -69, 54 | DAN | FPN |
| 129 | | | SPL_L_5_3 | A5l, lateral area 5 | -33, -47, 50 | DAN | FPN |
| 130 | | | SPL_R_5_3 | A5l, lateral area 5 | 35, -42, 54 | DAN | FPN |
| 131 | | | SPL_L_5_4 | A7pc, poAudentral area 7 | -22, -47, 65 | DAN | Unselected |
| 132 | | | SPL_R_5_4 | A7pc, poAudentral area 7 | 23, -43, 67 | DAN | Unselected |
| 133 | | | SPL_L_5_5 | A7ip, intraparietal area 7(hIP3) | -27, -59, 54 | DAN | FPN |
| 134 | | | SPL_R_5_5 | A7ip, intraparietal area 7(hIP3) | 31, -54, 53 | DAN | FPN |
| 135 | | IPL, Inferior Parietal Lobule | IPL_L_6_1 | A39c, caudal area 39(PGp) | -34, -80, 29 | DAN | Vis |
| 136 | | | IPL_R_6_1 | A39c, caudal area 39(PGp) | 45, -71, 20 | Unselected | Vis |
| 137 | | | IPL_L_6_2 | A39rd, rostrodorsal area 39(Hip3) | -38, -61, 46 | DMN | FPN |
| 138 | | | IPL_R_6_2 | A39rd, rostrodorsal area 39(Hip3) | 39, -65, 44 | FPN | FPN |
| 139 | | | IPL_L_6_3 | A40rd, rostrodorsal area 40(PFt) | -51, -33, 42 | DAN | FPN |
| 140 | | | IPL_R_6_3 | A40rd, rostrodorsal area 40(PFt) | 47, -35, 45 | DAN | FPN |
| 141 | | | IPL_L_6_4 | A40c, caudal area 40(PFm) | -56, -49, 38 | DMN | DMN |
| 142 | | | IPL_R_6_4 | A40c, caudal area 40(PFm) | 57, -44, 38 | FPN | FPN |
| 143 | | | IPL_L_6_5 | A39rv, rostroventral area 39(PGa) | -47, -65, 26 | DMN | DMN |
| 144 | | | IPL_R_6_5 | A39rv, rostroventral area 39(PGa) | 53, -54, 25 | DMN | DMN |
| 145 | | | IPL_L_6_6 | A40rv, rostroventral area 40(PFop) | -53, -31, 23 | Unselected | CO/SN |
| 146 | | | IPL_R_6_6 | A40rv, rostroventral area 40(PFop) | 55, -26, 26 | Unselected | CO/SN |
| 147 | | Pcun, Precuneus | PCun_L_4_1 | A7m, medial area 7(PEp) | -5, -63, 51 | DAN | Unselected |
| 148 | | | PCun_R_4_1 | A7m, medial area 7(PEp) | 6, -65, 51 | DAN | FPN |
| 149 | | | PCun_L_4_2 | A5m, medial area 5(PEm) | -8, -47, 57 | Unselected | Unselected |
| 150 | | | PCun_R_4_2 | A5m, medial area 5(PEm) | 7, -47, 58 | Unselected | Unselected |
| 151 | | | PCun_L_4_3 | dmPOS, dorsomedial parietooccipital sulcus(PEr) | -12, -67, 25 | Unselected | Prec |
| 152 | | | PCun_R_4_3 | dmPOS, dorsomedial parietooccipital sulcus(PEr) | 16, -64, 25 | Unselected | Prec |
| 153 | | | PCun_L_4_4 | A31, area 31 (Lc1) | -6, -55, 34 | DMN | DMN |
| 154 | | | PCun_R_4_4 | A31, area 31 (Lc1) | 6, -54, 35 | DMN | DMN |
| 155 | | PoG, Postcentral Gyrus | PoG_L_4_1 | A1/2/3ulhf, area 1/2/3(upper limb, head and face region) | -50, -16, 43 | SMN | SMN |
| 156 | | | PoG_R_4_1 | A1/2/3ulhf, area 1/2/3(upper limb, head and face region) | 50, -14, 44 | SMN | SMN |
| 157 | | | PoG_L_4_2 | A1/2/3tonIa, area 1/2/3(tongue and larynx region) | -56, -14, 16 | Unselected | CO/SN |
| 158 | | | PoG_R_4_2 | A1/2/3tonIa, area 1/2/3(tongue and larynx region) | 56, -10, 15 | Unselected | CO/SN |
| 159 | | | PoG_L_4_3 | A2, area 2 | -46, -30, 50 | Unselected | Unselected |
| 160 | | | PoG_R_4_3 | A2, area 2 | 48, -24, 48 | Unselected | Unselected |
| 161 | | | PoG_L_4_4 | A1/2/3tru, area1/2/3(trunk region) | -21, -35, 68 | SMN | SMN |
| 162 | | | PoG_R_4_4 | A1/2/3tru, area1/2/3(trunk region) | 20, -33, 69 | SMN | SMN |

| LabelID | Lobe | Gyrus | Name | Anatomical and modified Cyto-architectonic descriptions | MNI center of gravity (X,Y,Z) | Community Symbolic | Community Nonsymbolic |
|---|---|---|---|---|---|---|---|
| 163 | Insular Lobe | INS, Insular Gyrus | INS_L_6_1 | G, hypergranular insula | -36, -20, 10 | Unselected | CO/SN |
| 164 | | | INS_R_6_1 | G, hypergranular insula | 37, -18, 8 | Unselected | CO/SN |
| 165 | | | INS_L_6_2 | vIa, ventral agranular insula | -32, 14, -13 | DMN | DMN |
| 166 | | | INS_R_6_2 | vIa, ventral agranular insula | 33, 14, -13 | DMN | DMN |
| 167 | | | INS_L_6_3 | dIa, dorsal agranular insula | -34, 18, 1 | FPN | FPN |
| 168 | | | INS_R_6_3 | dIa, dorsal agranular insula | 36, 18, 1 | FPN | FPN |
| 169 | | | INS_L_6_4 | vId/vIg, ventral dysgranular and granular insula | -38, -4, -9 | Unselected | Unselected |
| 170 | | | INS_R_6_4 | vId/vIg, ventral dysgranular and granular insula | 39, -2, -9 | Unselected | Unselected |
| 171 | | | INS_L_6_5 | dIg, dorsal granular insula | -38, -8, 8 | Unselected | CO/SN |
| 172 | | | INS_R_6_5 | dIg, dorsal granular insula | 39, -7, 8 | Unselected | CO/SN |
| 173 | | | INS_L_6_6 | dId, dorsal dysgranular insula | -38, 5, 5 | Unselected | CO/SN |
| 174 | | | INS_R_6_6 | dId, dorsal dysgranular insula | 38, 5, 5 | Unselected | CO/SN |
| 175 | Limbic Lobe | CG, Cingulate Gyrus | CG_L_7_1 | A23d, dorsal area 23 | -4, -39, 31 | DMN | DMN |
| 176 | | | CG_R_7_1 | A23d, dorsal area 23 | 4, -37, 32 | DMN | DMN |
| 177 | | | CG_L_7_2 | A24rv, rostroventral area 24 | -3, 8, 25 | Unselected | Unselected |
| 178 | | | CG_R_7_2 | A24rv, rostroventral area 24 | 5, 22, 12 | DMN | DMN |
| 179 | | | CG_L_7_3 | A32p, pregenual area 32 | -6, 34, 21 | Unselected | DMN |
| 180 | | | CG_R_7_3 | A32p, pregenual area 32 | 5, 28, 27 | Unselected | Unselected |
| 181 | | | CG_L_7_4 | A23v, ventral area 23 | -8, -47, 10 | DMN | Prec |
| 182 | | | CG_R_7_4 | A23v, ventral area 23 | 9, -44, 11 | Unselected | Prec |
| 183 | | | CG_L_7_5 | A24cd, caudodorsal area 24 | -5, 7, 37 | Unselected | Unselected |
| 184 | | | CG_R_7_5 | A24cd, caudodorsal area 24 | 4, 6, 38 | Unselected | Unselected |
| 185 | | | CG_L_7_6 | A23c, caudal area 23 | -7, -23, 41 | Unselected | Unselected |
| 186 | | | CG_R_7_6 | A23c, caudal area 23 | 6, -20, 40 | Unselected | Unselected |
| 187 | | | CG_L_7_7 | A32sg, subgenual area 32 | -4, 39, -2 | DMN | DMN |
| 188 | | | CG_R_7_7 | A32sg, subgenual area 32 | 5, 41, 6 | DMN | DMN |
| 189 | Occipital Lobe | MVOcC, MedioVentral Occipital Cortex | MVOcC_L_5_1 | cLinG, caudal lingual gyrus | -11, -82, -11 | Unselected | Vis |
| 190 | | | MVOcC_R_5_1 | cLinG, caudal lingual gyrus | 10, -85, -9 | Vis | Vis |
| 191 | | | MVOcC_L_5_2 | rCunG, rostral cuneus gyrus | -5, -81, 10 | Vis | Vis |
| 192 | | | MVOcC_R_5_2 | rCunG, rostral cuneus gyrus | 7, -76, 11 | Vis | Vis |
| 193 | | | MVOcC_L_5_3 | cCunG, caudal cuneus gyrus | -6, -94, 1 | Vis | Vis |
| 194 | | | MVOcC_R_5_3 | cCunG, caudal cuneus gyrus | 8, -90, 12 | Vis | Vis |
| 195 | | | MVOcC_L_5_4 | rLinG, rostral lingual gyrus | -17, -60, -6 | Unselected | Vis |
| 196 | | | MVOcC_R_5_4 | rLinG, rostral lingual gyrus | 18, -60, -7 | Unselected | Vis |
| 197 | | | MVOcC_L_5_5 | vmPOS,ventromedial parietooccipital sulcus | -13, -68, 12 | Vis | Vis |
| 198 | | | MVOcC_R_5_5 | vmPOS,ventromedial parietooccipital sulcus | 15, -63, 12 | Vis | Vis |
| 199 | | LOcC, lateral Occipital Cortex | LOcC_L_4_1 | mOccG, middle occipital gyrus | -31, -89, 11 | Vis | Vis |
| 200 | | | LOcC_R_4_1 | mOccG, middle occipital gyrus | 34, -86, 11 | Vis | Vis |
| 201 | | | LOcC_L_4_2 | V5/MT+, area V5/MT+ | -46, -74, 3 | Vis | Vis |
| 202 | | | LOcC_R_4_2 | V5/MT+, area V5/MT+ | 48, -70, -1 | Vis | Vis |
| 203 | | | LOcC_L_4_3 | OPC, occipital polar cortex | -18, -99, 2 | Vis | Vis |
| 204 | | | LOcC_R_4_3 | OPC, occipital polar cortex | 22, -97, 4 | Vis | Vis |
| 205 | | | LOcC_L_4_4 | iOccG, inferior occipital gyrus | -30, -88, -12 | Vis | Vis |
| 206 | | | LOcC_R_4_4 | iOccG, inferior occipital gyrus | 32, -85, -12 | Vis | Vis |
| 207 | | | LOcC_L_2_1 | msOccG, medial superior occipital gyrus | -11, -88, 31 | Vis | Vis |
| 208 | | | LOcC_R_2_1 | msOccG, medial superior occipital gyrus | 16, -85, 34 | Vis | Vis |
| 209 | | | LOcC_L_2_2 | lsOccG, lateral superior occipital gyrus | -22, -77, 36 | Vis | Vis |
| 210 | | | LOcC_R_2_2 | lsOccG, lateral superior occipital gyrus | 29, -75, 36 | DAN | Vis |
| 211 | Subcortical Nuclei | Amyg, Amygdala | Amyg_L_2_1 | mAmyg, medial amygdala | -19, -2, -20 | Dropout | Dropout |
| 212 | | | Amyg_R_2_1 | mAmyg, medial amygdala | 19, -2, -19 | Dropout | Dropout |
| 213 | | | Amyg_L_2_2 | lAmyg, lateral amygdala | -27, -4, -20 | Dropout | Dropout |
| 214 | | | Amyg_R_2_2 | lAmyg, lateral amygdala | 28, -3, -20 | Hipp | Hipp |
| 215 | | Hipp, Hippocampus | Hipp_L_2_1 | rHipp, rostral hippocampus | -22, -14, -19 | Hipp | Hipp |
| 216 | | | Hipp_R_2_1 | rHipp, rostral hippocampus | 22, -12, -20 | Dropout | Dropout |
| 217 | | | Hipp_L_2_2 | cHipp, caudal hippocampus | -28, -30, -10 | Hipp | Hipp |
| 218 | | | Hipp_R_2_2 | cHipp, caudal hippocampus | 29, -27, -10 | Hipp | Hipp |
| 219 | | BG, Basal Ganglia | BG_L_6_1 | vCa, ventral caudate | -12, 14, 0 | CdV/NAc | BG |
| 220 | | | BG_R_6_1 | vCa, ventral caudate | 15, 14, -2 | CdV/NAc | BG |
| 221 | | | BG_L_6_2 | GP, globus pallidus | -22, -2, 4 | BG | BG |
| 222 | | | BG_R_6_2 | GP, globus pallidus | 22, -2, 3 | BG | BG |
| 223 | | | BG_L_6_3 | NAC, nucleus accumbens | -17, 3, -9 | CdV/NAc | BG |
| 224 | | | BG_R_6_3 | NAC, nucleus accumbens | 15, 8, -9 | CdV/NAc | BG |
| 225 | | | BG_L_6_4 | vmPu, ventromedial putamen | -23, 7, -4 | BG | BG |
| 226 | | | BG_R_6_4 | vmPu, ventromedial putamen | 22, 8, -1 | BG | BG |
| 227 | | | BG_L_6_5 | dCa, dorsal caudate | -14, 2, 16 | FPN | CdD |
| 228 | | | BG_R_6_5 | dCa, dorsal caudate | 14, 5, 14 | FPN | CdD |
| 229 | | | BG_L_6_6 | dlPu, dorsolateral putamen | -28, -5, 2 | BG | BG |
| 230 | | | BG_R_6_6 | dlPu, dorsolateral putamen | 29, -3, 1 | BG | BG |
| 231 | | Tha, Thalamus | Tha_L_8_1 | mPFtha, medial pre-frontal thalamus | -7, -12, 5 | Thal | Thal |
| 232 | | | Tha_R_8_1 | mPFtha, medial pre-frontal thalamus | 7, -11, 6 | Thal | Thal |
| 233 | | | Tha_L_8_2 | mPMtha, pre-motor thalamus | -18, -13, 3 | Thal | Thal |
| 234 | | | Tha_R_8_2 | mPMtha, pre-motor thalamus | 12, -14, 1 | Thal | Thal |
| 235 | | | Tha_L_8_3 | Stha, sensory thalamus | -18, -23, 4 | Thal | Thal |
| 236 | | | Tha_R_8_3 | Stha, sensory thalamus | 18, -22, 3 | Thal | Thal |
| 237 | | | Tha_L_8_4 | rTtha, rostral temporal thalamus | -7, -14, 7 | Thal | Thal |
| 238 | | | Tha_R_8_4 | rTtha, rostral temporal thalamus | 3, -13, 5 | Thal | Thal |
| 239 | | | Tha_L_8_5 | PPtha, posterior parietal thalamus | -16, -24, 6 | Thal | Thal |
| 240 | | | Tha_R_8_5 | PPtha, posterior parietal thalamus | 15, -25, 6 | Thal | Thal |
| 241 | | | Tha_L_8_6 | Otha, occipital thalamus | -15, -28, 4 | Thal | Thal |
| 242 | | | Tha_R_8_6 | Otha, occipital thalamus | 13, -27, 8 | Thal | Thal |
| 243 | | | Tha_L_8_7 | cTtha, caudal temporal thalamus | -12, -22, 13 | Thal | Thal |
| 244 | | | Tha_R_8_7 | cTtha, caudal temporal thalamus | 10, -14, 14 | Thal | Thal |
| 245 | | | Tha_L_8_8 | lPFtha, lateral pre-frontal thalamus | -11, -14, 2 | Thal | Thal |
| 246 | | | Tha_R_8_8 | lPFtha, lateral pre-frontal thalamus | 13, -16, 7 | Thal | Thal |

**Supplementary Table S1 – Region table with community assignments.** All 246 regions of the Brainnetome atlas (Fan et al., 2016) are listed, with information including their lobe, gyrus, anatomic description and center of gravity coordinates in MNI space. Colored coded community assignments from the symbolic and nonsymbolic conditions are indicated in the rightmost columns. Unselected = region was assigned to community which did not reach our criteria for selection based on a significant modularity contribution at the subject-level (see Supplementary Fig S1 and Supplementary Methods); Dropout = region determined to suffer from signal dropout in at least one subject (see Supplementary Methods); DMN = Default mode network; Thal = Thalamus; FPN = Frontoparietal network; Vis = Visual; DAN = Dorsal attention network; BG = Basal ganglia; SMN = Sensorimotor network; Hipp = Hippocampus; CdV/NAc = Ventral caudate/Nucleus accumbens; Aud = Auditory; Prec = Precuneus; CO/SN = Cingulo-opercular/Salience network; CdD = Dorsal caudate; ns = community not selected.

# References

Betzel, R. F., Medaglia, J. D., Papadopoulos, L., Baum, G. L., Gur, R., Gur, R., … Bassett, D. S. (2017). The modular organization of human anatomical brain networks: Accounting for the cost of wiring. *Network Neuroscience*, *1*(1), 42–68. https://doi.org/10.1162/NETN_a_00002

Fan, L., Li, H., Zhuo, J., Zhang, Y., Wang, J., Chen, L., … Jiang, T. (2016). The Human Brainnetome Atlas: A New Brain Atlas Based on Connectional Architecture. *Cerebral Cortex*, *26*(8), 3508–3526. https://doi.org/10.1093/cercor/bhw157

Geerligs, L., Rubinov, M., Cam-CAN, & Henson, R. N. (2015). State and Trait Components of Functional Connectivity: Individual Differences Vary with Mental State. *Journal of Neuroscience*, *35*(41), 13949–13961. https://doi.org/10.1523/JNEUROSCI.1324-15.2015

Leys, C., Ley, C., Klein, O., Bernard, P., & Licata, L. (2013). Detecting outliers: Do not use standard deviation around the mean, use absolute deviation around the median. *Journal of Experimental Social Psychology*, *49*(4), 764–766. https://doi.org/10.1016/j.jesp.2013.03.013

Peer, M., Abboud, S., Hertz, U., Amedi, A., & Arzy, S. (2016). Intensity-based masking: A tool to improve functional connectivity results of resting-state fMRI. *Human Brain Mapping*, *37*(7), 2407–2418. https://doi.org/10.1002/hbm.23182
